# In silico molecular docking and molecular dynamic simulation of agarwood compounds with molecular targets of Alzheimer’s disease 

**DOI:** 10.12688/f1000research.130618.1

**Published:** 2023-03-01

**Authors:** Phaniendra Alugoju, Vishwambar Vishnu Bhandare, Vishal S. Patil, Krishna Swamy V. K. D, Prem Kumar Borugadda, Tewin Tencomnao

**Affiliations:** 1 Department of Clinical Chemistry, Faculty of Allied Health Sciences, Chulalongkorn University, Bangkok, 10330, Thailand; 2 Natural Products for Neuroprotection and Anti-Ageing Research Unit, Chulalongkorn University, Bangkok, 10330, Thailand; 3 Department of Microbiology, Shivaji University, Kolhapur, Maharashtra, 416004, India; 4 Department of Pharmacology and Toxicology, KLE College of Pharmacy Belagavi, KLE Academy of Higher Education and Research (KAHER), Belagavi, Karnataka, 590010, India; 5 ICMR-National Institute of Traditional Medicine, Belagavi, Karnataka, 590010, India; 6 Phytomedicine and Ageing laboratory, Department of Biochemistry and Molecular Biology, Pondicherry University, Puducherry, Puducherry, 605014, India; 7 Department of Computer Science, School of Engineering and Technology, Pondicherry University (A Central University), Karaikal Campus, Karaikal, Puducherry, 609605, India

**Keywords:** agarwood, neurodegeneration, docking, molecular dynamic simulation.

## Abstract

Background

Alzheimer's disease (AD) is a neurological condition that primarily affects older people. Currently available AD drugs are associated with side effects and there is a need to develop natural drugs from plants. Aquilaria is as an endangered medicinal plant genus (commonly called agarwood plants) and various products of Aquilaria plant spp. including resinous heartwood, leaves, bark, and stem have been widely used in various traditional medicine systems. Research on agarwood plants is sparse and only a few previous studies demonstrated their neuroprotective properties *in vitro.* Owing to the presence of a plethora of secondary metabolites in agarwood plants, it is imperative not only to protect these plants but also evaluate the bioactivity of agarwood phytochemicals.

Methods

Computational methods such as AutoDock Vina and molecular dynamic (MD) simulations were employed for the docking of 41 selected agarwood compounds with AD-related molecular targets.

Results and Conclusion

According to docking data, three compounds aquilarisin, aquilarisinin, aquilarixanthone showed highest binding affinity to selected AD targets compared to their known inhibitors. MD simulation studies revealed that, selected agarwood compounds' protein-ligand complexes showed remarkable structural stability throughout 100 ns simulation. The agarwood chemicals aquilarisin, aquilarisinin, aquilarixanthone, pillion, and agarotetrol are consequently suggested as some of the found hits against AD targets, however, additional experimental validation is required to establish their effectiveness.

## Introduction

Alzheimer's disease (AD) makes up 75% of instances of dementia and is the most common neurodegenerative ailment affecting those over 65 years. ^1^^–^^3^ Extracellular amyloid plaques, also known as “Aβ plaques,“ or “senile plaques,“ rich in amyloid-(Aβ), and intracellular neurofibrillary tangles (NFTs), rich in tau proteins, are the two key pathological hallmarks of AD. ^4^ Several proteins associated with the neurological dysfunction in AD include cholinesterases, ^5^ N-methyl D-aspartate (NMDA) receptor, ^6^ beta-site amyloid precursor protein cleaving enzyme 1 (BACE 1), ^7^ Asparagine endopeptidase (AEP), ^8^ Monoamine oxidases (MAO) ^9^ and protein kinases. ^10^^,^^11^ AD has become a global health problem due to the lack of effective treatment for the amelioration of neurological dysfunction. ^12^ The current pharmacologically important AD drugs include cholinesterase inhibitors such as donepezil, galantamine, and rivastigmine block the breakdown of acetylcholine, thereby increasing acetylcholine (Ach) levels in brain and help in improving cognitive function. ^3^ Galantamine is the only naturally occurring inhibitor belonging to alkaloid class of phytochemicals and it can reversibly and competitively inhibit *acetylcholinesterase* (AChE). Memantine is the currently available NMDA receptor antagonist that can improve cognition and memory problems in AD by balancing the glutamatergic system. ^6^ However, some side effects of these medications include nausea, headache, vomiting, and dizziness. Therefore, there has been a great interest in identifying potent natural inhibitors of target proteins of AD. ^7^

Since ancient times, traditional medicinal plants have been utilized as a major source of drugs to treat various kinds of human illnesses including neurological disorders. ^13^ Natural phytochemicals have received much attention in recent years due to their pharmacophore-like structures and pharmacokinetic properties. Owing to the presence of a plethora of phytochemicals in medicinal plants, the systematic analysis of each phytochemical by conventional methods is cumbersome and a time taking process. ^14^ In this context, the computer aided drug design techniques have been widely used for the screening of chemical libraries and identification of molecular targets of natural or synthetic compounds. ^15^ Virtual screening is considered as the standard initial step in the drug discovery process prior to wet lab experiments. ^15^ *In silico* approaches significantly increased the effectiveness of assessing the bioactive compounds of medicinal plants. In fact, using *in silico* approaches, some Food and Drug Administration (FDA) approved drugs were developed. Several studies have performed *in silico* analysis on phytochemicals of medicinal plants against target proteins of human diseases. ^16^^–^^20^ Previous studies have used computational tools to identify and predict possible anti-Alzheimer’s potential of bioactive compounds in medicinal plants. ^17^^,^^21^^–^^23^

Aquilaria is an endangered medicinal plant genus that is currently protected by international laws due to indiscriminate cutting for various commercial, cultural, religious, and medicinal purposes. Aquilaria spp. trees, commonly known as agarwood, are primarily found in Southeast Asia. The products of Aquilaria spp, including agarwood, leaves, bark, stem etc., have been extensively used in Asia for the treatment of a variety of ailments such as cough, pain, and allergy. Agarwood is a valuable, non-timber, resinous portion that is used for making incense, perfume, cosmetics, and personal care products, as well as for the production of traditional Ayurvedic, Chinese, Thai, Korean, Tibetan, and Eastern medicines for curing many ailments such as arthritis, inflammation, diarrhoea, and used as a soporific, antidepressant, and cardio-protectant. ^24^ Other plant materials of Aquilaria spp. including leaves, stem, and bark have been found to have several pharmacological properties such as anallergic, cytotoxic, anti-inflammatory, cardioprotective, antimicrobial, anti-oxidant, hepatoprotective, laxative, and mosquitocidal effect. ^25^^,^^26^ Compounds extracted from the resinous heartwood of *Aquilaria sinensis* showed notable neuroprotective effects on corticosterone and 1-methyl-4-phenylpyridinium (MPP ^+^)-induced injury in PC12 cells ^27^^,^^28^ and also exhibited obvious cytotoxic activity. ^29^ The benzene extractable compounds of agarwood (jinkoh-eremol and agarospirol) *Aquilaria malaccensis* possess potent anti-depressant and anti-psychotic activities. ^30^^,^^31^ The chloroform extracts of the leaves and stem of *Aquilaria subintegra* showed significant AChE inhibitory activity. ^32^ Compounds isolated from *Aquilaria crassna* leaves were also shown to exhibit neuritogenic properties and therefore exerted neuroprotective effects in P19-derived neurons. ^33^ *A. crassna* leaf extracts have been demonstrated to ameliorate glucose-Induced neurotoxicity *in vitro.* ^34^ Our unpublished experimental data also indicate that both leaf and agarwood extracts of *A. crassna* can exert protective effects against D-galactose induced neurotoxicity in mouse hippocampal HT-22 cell line.

Traditional understanding of herbal supplements is helpful for developing cutting-edge drugs for a number of illnesses, including neurological disorders like Alzheimer's disease (AD). ^13^ There is still a lack of research on agarwood plants due to extreme demand and depletion of natural resources. ^35^ However, neuroprotective activity of these agarwood plants is poorly explored. Growing evidence suggests the use of *in silico* studies as the first step before setting up *in vitro* or *in vivo* experiments. Thus, virtual screening of small molecules library with known AD targets is critical for identification and subsequent validation of best possible hits in either cell lines or animal models. Hence in the present study, molecular docking of selected agarwood compounds from PubChem with molecular target proteins of AD was performed for the first time. Structural stability of the best docked agarwood compounds with AD targets has been studied using molecular dynamic (MD) simulations.

## Methods

### Selection and Preparation of ligands

**Figure 1.**
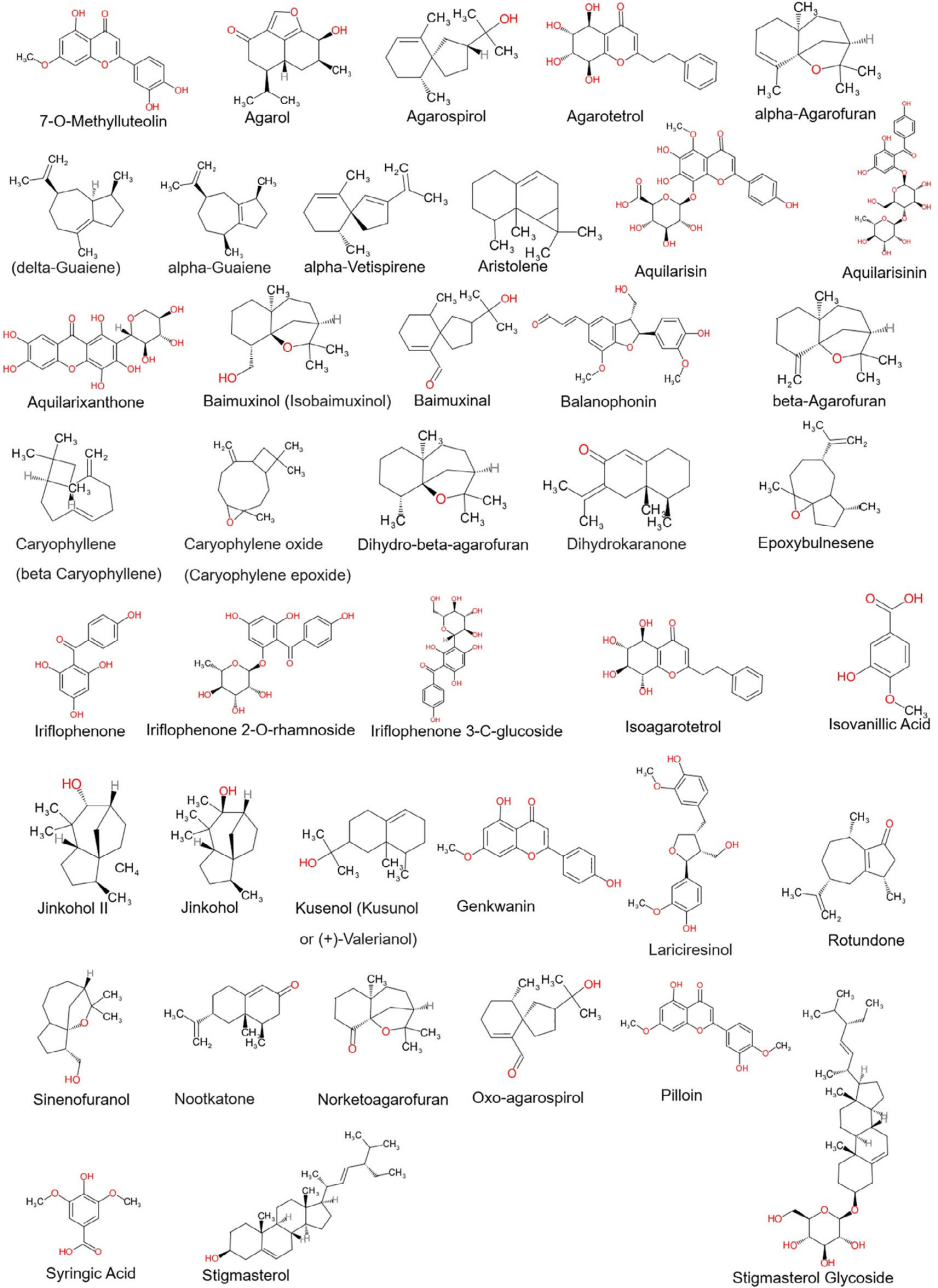
2D structures of agarwood phytocompounds chosen.

Phytocompounds from the Aquilaria plant species were selected based on the previous literature and their structures were retrieved from the PubChem database (refer Figure 1). The phytocompounds structures were prepared by adding polar hydrogens, Gasteiger charges and by performing energy minimization in UCSF Chimera 1.16 using default parameters. ^36^

### Prediction of physicochemical properties

Prediction of pharmacokinetic and pharmacodynamic features can be accomplished by using the physicochemical characteristics of chemical substances, such as lipophilicity (LogP), solubility (LogS), and polar surface area and volume (PSA). ^37^ It is crucial to analyze these features because they affect how they interact with transport proteins and enzymes that are involved in drug clearance. ^37^ In the present study, we have used Molsoft tools ( https://www.molsoft.com/mprop/) to predict the number of H-bond donors (No. of HBD) and acceptors (No. of HBA) present, polar surface area (MolPSA), lipophilicity (Mol LogP), solubility (Mol LogP), and the molecular polar surface area and volume (Mol PSA) of tested ligands. ^38^ Also, ADMET properties such as absorption, digestion, metabolism, excretion, and toxicity properties of selected ligands were predicted using ADMETlab 2.0 ( https://admetmesh.scbdd.com/).

### Selection and Preparation of receptors

The crystal structures of different proteins implicated in the pathogenesis of AD were retrieved from RCSB PDB structural database ( https://www.rcsb.org/). The stereo-chemical properties, Ramachandran graph and values of selected proteins were evaluated by Molprobity server. ^39^ Chimera 1.16 (RRID:SCR_004097) was used to generate any missing residues in the selected target proteins. Following the removal of unneeded nonstandard heteroatoms, polar hydrogens and Gasteiger charges were added. All targets' structural details were refined using the steepest descent and conjugate gradient algorithms (100 steps each) with amber force field (Amber ff14SB). ^40^ Then, using AutoDock tools 1.5.7 (RRID:SCR_012746), the energy-minimized protein structures were transformed into ‘ *pdbqt*’ format. A list of proteins along with their PDB IDs are given in Table 1.

**Table 1.** List of selected target proteins associated with AD.

PDB ID	Protein Name	X-ray resolution (Å)
1PBQ	NR1 Ligand Binding Core in Complex With 5,7-Dichlorokynurenic Acid	1.90
1Q5K	Human glycogen synthase kinase 3	1.94
1UDT	Human phosphodiesterase-5 (pd-5)	2.30
2Y9Q	human ERK2 complexed with a MAPK docking peptide	1.55
2Z5X	Human MAO-A	2.20
4AU8	Human cdk5	1.90
4BDS	Human butyrylcholinesterase	2.10
4KKE	Human AMP-bound JNK3	2.20
4M0F	Human Acetylcholinesterase	2.30
5BTR	Human SIRT1	3.20
5IE1	Human BAEC1	2.30
5LUA	human legumain (AEP)	2.00
6GZM	Human CASEIN KINASE1 delta	1.59

### Protein-ligand docking

Docking was performed with Autodock Vina as described in our previous study. ^41^ The grid box's dimensions were fixed at XYZ=30Å×30Å×30Å which was found to be the best size for the default exhaustiveness (=8), and the ligand binding site was positioned in the middle of the grid box. The spatial dimension (XYZ axis) and the grid box’s size were specified in a configuration file. Using AutoDock vina version 1.1.2's (RRID:SCR_011958) command line interface, docking was accomplished. The obtained results are restricted to nine binding modes. The log file created included a list of the increasing binding modes and their associated binding energies. The BIOVIA Discovery studio visualizer 2021 was used to view the binding modes. All non-bonded interactions were recorded (DOI: dx.doi.org/10.17504/protocols.io.3byl4j362lo5/v1).

### Molecular dynamic (MD) simulation

MD simulation using Gromacs 2020.5 (RRID:SCR_014565) was used to track the structural stability of the docked complexes. Gromos96 force field was used to create the topology of the protein, and PRODRG server ( http://davapc1.bioch.dundee.ac.uk/cgi-bin/prodrg/submit.html) was utilized to create the topology of the ligand. The docked complexes were solvated in a cuboidal box with adequate size to fit the complete complex in the middle using a “Simple Point Charge“ (SPC) water model. Appropriate number of counter ions (Na+/Cl-) were used to neutralize the simulated systems. The undesirable contacts and steric conflicts were then removed from the neutralized systems using steepest descent followed by conjugate gradient methods for 50,000 steps each.

The NVT ensemble used to maintain constant number of atoms, volume, and temperature, further NPT ensemble was used to maintain constant pressure. In this study, we set temperature and pressure constant at 300K and 1 bar respectively. Further, followed by 1ns of equilibration, unrestrained MD simulation was performed for a period of 100ns in solvent. The Particle Mesh Ewald (PME) method was used to handle coulomb electrostatic interactions, while the LINear Constraint Solver (LINCS) algorithm was used to limit H-bonds. Using a cut-off value of 14 Å, the non-bonded contacts were trimmed. The trajectories generated were analyzed using some of the inbuild gromacs tools like ‘ *gmx rms’*, ‘ *gmx rmsf’*, ‘ *gmx hbond’*, ‘ *gmx gyrate’*, ‘ *gmx sasa’*, etc. and other additional packages for specific analysis wherever required. Conformational changes at the secondary structural level were monitored by using Dictionary of Protein Secondary Structure (DSSP) software (RRID:SCR_002725) (DOI: dx.doi.org/10.17504/protocols.io.36wgqjmp5vk5/v1)

### Molecular mechanic/Poisson-Boltzmann surface area (MM-PBSA) calculation

MM-PBSA in conjunction with MD simulations is commonly used to determine the binding free energy of protein and ligand complexes. ^42^ It uses the following equation as:

$$∆G_{\text{Binding}}=G_{\text{Complex}}-\;G_{\text{Receptor}}-G_{\text{Ligand}}$$

where G _Complex_ is total free energy of the ligand-protein complex, G _Receptor_ and G _Ligand_ are total free energies of the isolated protein and ligand in the solvent, respectively. The “ *g_mmpbsa*” tool ^43^ was used to calculate binding free energy using the MM-PBSA method. For the binding free energy estimate, the stable trajectory seen between 50 and 100 ns was selected.

### Enrichment analysis of protein targets

In STRING database (version 11.0), the proteins Glycogen Synthase Kinase 3 Beta (GSK3B), Monoamine oxidase-A (MAOA), Butyrylcholinesterase (BChE), Acetylcholinesterase (AChE), beta-site amyloid precursor protein cleaving enzyme 1 (BACE1), Legumain (LGMN), and Glutamate Ionotropic Receptor NMDA type subunit 1 (GRIN1) were searched for *Homo sapiens.* Only these proteins were selected for enrichment analysis, because most of the tested ligands showed good binding affinity only towards them. Gene ontology (GO) analysis was used to pinpoint the biological processes. Based on the available literature, the pathways contributing to AD pathogenesis were selected.

### Network pharmacology

Cytoscape tool (version 3.9.1) was used to build the integrated network between chemicals, protein targets, and regulated processes. In the network analyser, the entire network was treated as “Direct” during network construction. The resulting network was inspected using a topological parameter “edge count. ^44^” The node size and colour were set using low values to small sizes and low values to bright colours, respectively. ^45^

## Results

### Prediction of ADMET analysis

The physicochemical properties of all the chosen agarwood compounds were studied to gain more structural features of the individual phytocompounds ( Table 2). Also, pharmacodynamic and pharmacokinetic properties of these compounds were analyzed using ADMETLab2.0. The analysis reveals that all the chosen compounds were less/not toxic. Additionally, it is interesting to note that most of the compounds had the capacity to cross the blood brain barrier (BBB). Though three phytocompounds namely, aquilarisin, aquilarisinin, and aquilarixanthone failed the Lipinski rule, they were shown to cross the blood brain barrier effectively and express lesser or no toxicity (Supplementary Material ADMET properties of agarwood compounds.xlsx). The blood brain barrier penetration values for agarotetrol, aquilarisin, aquilarisinin, aquilarixanthone, and pillion were predicted to be 0.8, 0.029, 0.35, 0.011, and 0.01, respectively. All the chosen ligands were predicted as non-carcinogenic and showed drug-likeliness.

**Table 2.** Physicochemical properties of Aquilaria compounds used in this study.

Ligand name	Mol Formula	Mol. Weight	No of HBA	No of HBD	Mol LogP ^c^	Mol LogS ^d^	MolPSA (Å ^2^)	No. of stereo centers
7-O-Methylluteolin	C16 H12 O6	300.06	6	3	3.21	-3.18	78.97	0
Agarol	C15 H20 O3	248.14	3	1	2.60	-2.69	38.63	4
Agarospirol	C15 H26 O	222.20	1	1	3.90	-3.94	16.44	3
Agarotetrol	C17H18O6	318.11	6	4	0.30	-1.25	84.74	4
Alpha-agarofuran	C15 H24 O	220.18	1	0	4.16	-4.27	7.63	3
Alpha-bulnesene	C15 H24	204.19	0	0	5.21	-5.05	0.00	3
Alpha-guaiene	C15 H24	204.19	0	0	4.93	-4.73	0.00	3
Alpha-vetispirene	C15 H22	202.17	0	0	4.37	-4.26	0.00	2
Aquilarisin	C22 H20 O13	492.09	13	7	1.25	-1.77	168.84	5
Aquilarisinin	C25 H30 O14	554.16	14	9	-0.46	-1.85	191.87	10
Aquilarixanthone	C18 H16 O11	408.07	11	8	0.18	-1.47	154.94	4
Aristolene	C15 H24	204.19	0	0	4.64	-4.24	0.00	4
Baimuxinal	C15 H24 O2	236.18	2	1	3.25	-2.70	30.60	3
baimuxinol	C15 H26 O2	238.19	2	1	3.26	-2.80	24.74	4
Balanophonin	C20 H20 O6	356.13	6	2	2.05	-2.44	70.65	2
Beta-agarofuran	C15 H24 O	220.18	1	0	3.92	-4.03	7.63	3
Beta-caryophyllene	C15 H24	204.19	0	0	5.35	-5.39	0.00	2
Caryophyllene epoxide	C15 H24 O	220.18	1	0	3.97	-4.30	8.34	4
Delta-guaiene	C15 H24	204.19	0	0	5.21	-5.05	0.00	3
Dihydro-beta-agarofuran	C15 H26 O	222.20	1	0	4.23	-4.19	7.63	4
Dihydrokaranone	C15 H22 O	218.17	1	0	4.18	-4.50	13.60	2
Epoxybulnesene	C15 H24 O	220.18	1	0	4.43	-4.33	8.03	5
genkwanin	C16 H12 O5	284.07	5	2	3.64	-3.58	63.49	0
Iriflophenone 2-O-rhamnoside	C19 H20 O9	392.11	9	6	0.56	-1.84	128.12	5
Iriflophenone 3-C-glucoside	C19 H20 O10	408.11	10	8	-0.12	-1.78	151.91	5
Irioflophenone	C13 H10 O5	246.05	5	4	2.00	-2.39	82.04	0
Isoagarotetrol	C17 H18 O6	318.11	6	4	0.30	-1.25	84.74	4
Isovanillic acid	C8 H8 O4	168.04	4	2	1.55	-1.82	52.83	0
Jinkohol II	C14 H24 O	208.18	1	1	3.41	-2.86	16.51	5
Jinkohol	C15 H26 O	222.20	1	1	3.87	-3.90	16.22	5
Kusenol	C15 H26 O	222.20	1	1	3.98	-4.25	16.07	3
Lariciresinol	C20 H24 O6	360.16	6	3	1.83	-1.89	74.69	3
Nookatone	C15 H22 O	218.17	1	0	3.90	-4.31	13.70	3
Norketoagarofuran	C14 H22 O2	222.16	2	0	3.10	-2.76	20.88	3
Oxo-agarospirol	C15 H24 O2	236.18	2	1	3.25	-2.70	30.60	3
pilloin	C17 H14 O6	314.08	6	2	3.71	-3.65	70.06	0
(-)-Rotundone	C15 H22 O	218.17	1	0	4.12	-3.91	14.30	3
Sinenofuranol	C14 H24 O2	224.18	2	1	2.76	-2.37	25.44	4
Stigmasterol glycoside	C35 H58 O6	574.42	6	4	5.60	-5.78	79.93	14
Stigmasterol	C29 H48 O	412.37	1	1	7.74	-6.24	16.28	9
Syringic acid	C9 H10 O5	198.05	5	2	0.82	-1.09	59.39	0

### Molecular docking analysis

**Figure 2.**
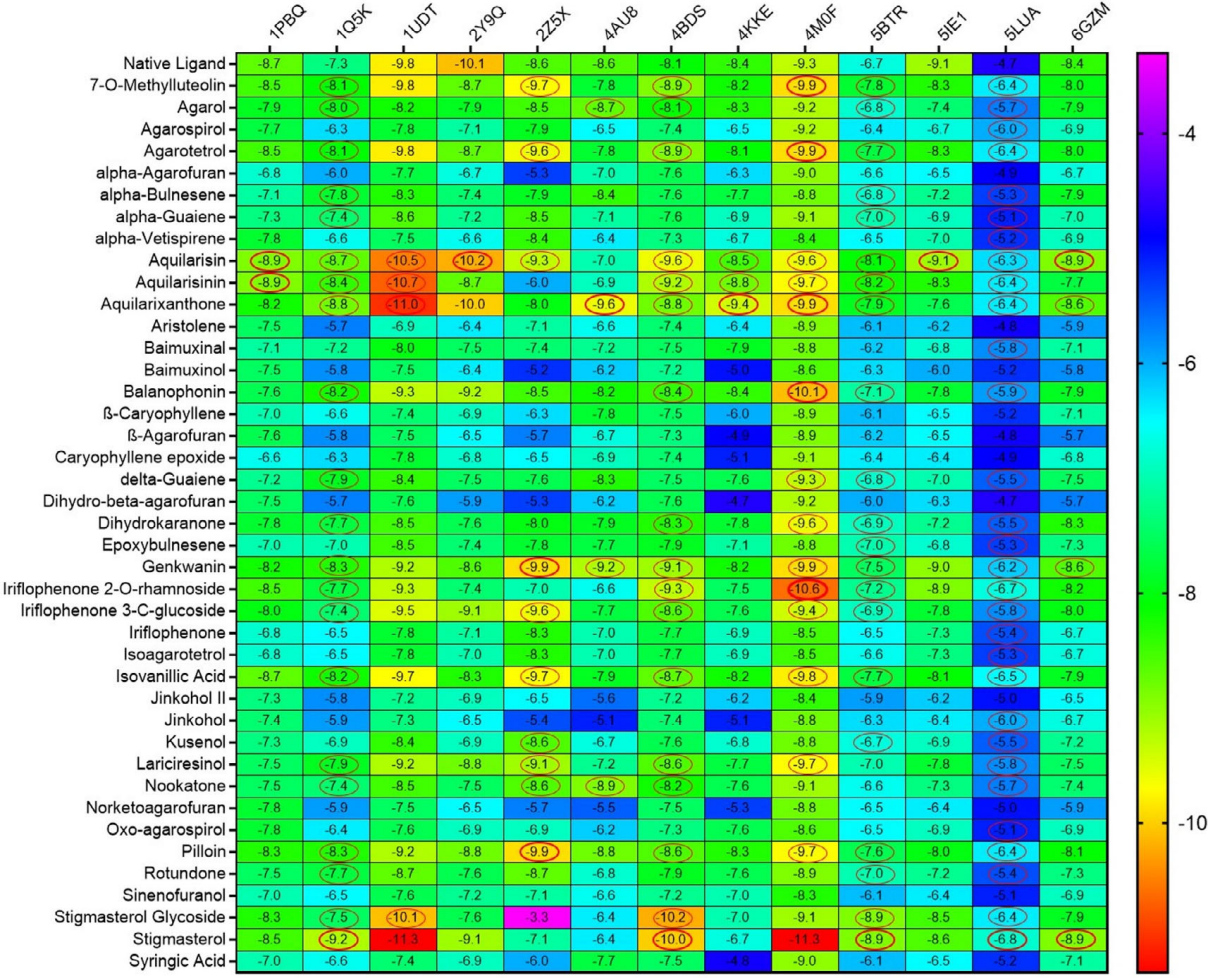
The heatmap represents the binding energies (kcal/mol) of the agarwood compounds docked with target protein of AD. Ligands with higher binding energies than the respective native ligands of target proteins are denoted in red circles.

In this study, docking of 41 agarwood compounds was done against 13 target proteins of AD. The selection of specific AD targets has been made considering their close and direct association with AD pathogenesis and its disease progression. Figure 2 represents heatmap of the binding energy estimated from the docking pose of agarwood compounds towards the tested molecular targets of AD. The scale of heat map ranges from least (blue) to highest binding (red) affinity was predicted based on the docking results. The binding interactions of only those ligands with least binding energy than the respective native ligands of AD target proteins are highlighted by circle in the heatmap (refer Figure 2). Ligands such as aquilarisin, aquilarisinin, and aquilarixanthone showed good binding affinity with most of the selected AD related proteins. Aquilarisin showied good binding affinity towards 12 of the 13 tested AD related proteins except with 4AU8. Aquilarisinin exhibited the highest binding affinity towards 8 of the 13 selected AD proteins including 1PBQ, 1Q5K, 1UDT, 4BDS, 4KKE, 4M0F, 5BTR, and 5LUA. On the other hand, aquilarixanthone was found to show highest binding affinity towards 9 of the 13 selected AD proteins including 1Q5K, 1UDT, 4AU8, 4BDS, 4KKE, 4M0F, 5BTR, 5LUA, and 6GZM.

**Figure 3.**
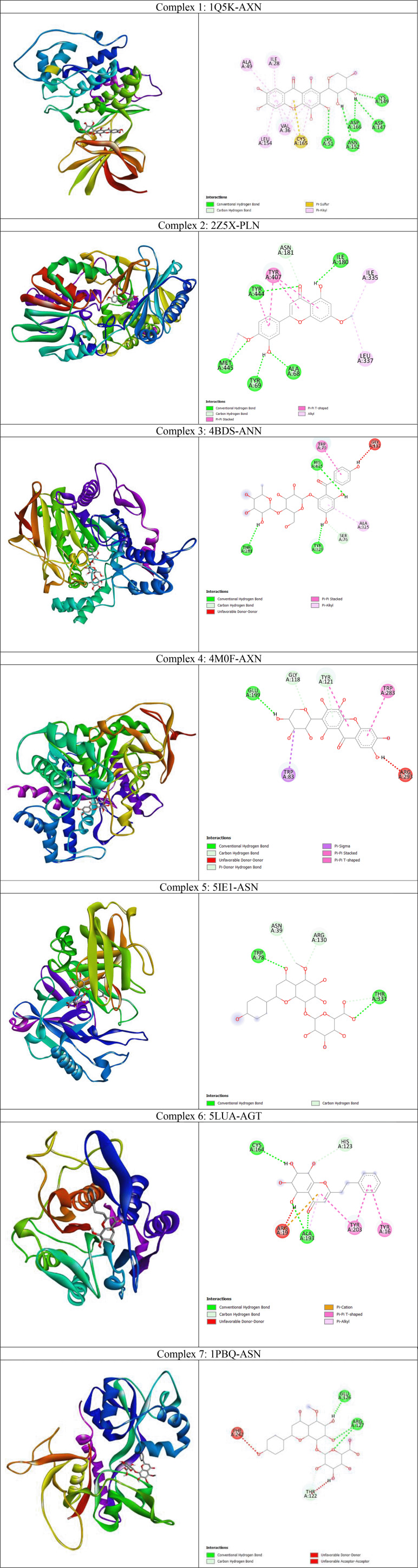
Binding mode of the Agarwood phytocompounds to the AD targets (shown in 3D) predicted by docking study and their intermolecular interactions stabilizing the docked complexes in 2D.

From the heatmap it was clear that proteins such as 1Q5K (GSK3beta), 2Z5X (MAO-B), 4BDS (BChE), 4M0F (AChE), 5BTR, 5LUA (AEP) formed best docked complexes with most of the selected agarwood compounds. The site-specific non-bonded interactions of some of the representative top docked complexes showing highest binding affinity and conserved binding pocket interactions are listed in Table 3 (and also the 3D and 2D structures of top docked complexes are shown in Figure 3). Further, to gain more detailed insights to their structural stability and intermolecular interactions we selected some of the best docked representative complexes for MD simulations. The phytocompounds bound to these selected targets had been chosen considering binding energy, H-bonds and other nonbonded interactions including hydrophobic and electrostatic interactions. Additionally conserved binding pocket interactions had also been considered and compared with the respective known control inhibitors for the respective targets. Thus, we selected total seven complexes namely 1Q5K-AXN (complex1), 2Z5X-PLN (complex2), 4BDS-ANN (Complex 3), 4M0F-AXN (Complex 4), 5IE1-ASN (Complex 5), 5LUA-AGT (Complex 6), and 1PBQ-ASN (Complex 7) for MD simulations. It was observed that the agarwood phytocompounds bind to the AD targets to form stable complexes, and these complexes are maintained through H-bonds and other non-bonded interactions (as shown in Table 3).

**Table 3.** The intermolecular interaction of best docked complexes.

		Amino acid residues involved in the bond formation			
PDB ID	Native/test Ligand (binding energy)	H-bond	H-bond length (Å)	No of H-bonds	Hydrophobic contacts
**1PBQ**	DK1 (-8.7)	ARG127	1.84	5	PHE88, PHE88, PRO120, VAL223, PHE12, TRP219, TRP219, PHE246, PRO120
		THR122	1.94		
		ARG127	2.29		
		PRO120	2.26		
		LEU121	2.67		
	ASN (-8.9)	ARG127	2.72	5	-
		ARG127	2.27		
		ARG127	2.65		
		GLU126	1.87		
		THR122	2.43		
	ANN (-8.9)	GLN91	2.25	11	LEU142
		ARG127	2.74		
		GLN140	2.90		
		SER176	2.78		
		SER244	2.96		
		GLU92	2.72		
		GLN91	2.51		
		GLU126	2.22		
		THR90	2.76		
		SER244	2.98		
		GLU126	3.45		
**1Q5K**	TMU	ASP166	2.53	3	ILE28, VAL36, ALA49, LEU154, CYS165, ILE28
		VAL101	2.06		
		VAL27	3.54		
	AGT	LYS51	2.50	4	VAL36, CYS165, ILE28, VAL36, ALA49, LEU154
		ASN152	2.77		
		GLN151	2.54		
		LYS51	2.49		
	ASN	VAL101	2.99	6	-
		ASP166	3.36245		
		GLY29	2.32		
		ARG107	2.94		
		ARG107	2.37		
		TYR100	3.47		
	ANN	SER32	1.88	6	VAL36
		LYS149	2.87		
		GLY31	2.66		
		GLY31	2.97		
		ASP166	3.03		
		ASP166	3.57		
	AXN	LYS51	2.44	7	CYS165, VAL36, VAL36, CYS165, ILE28, VAL36, ALA49, LEU154, CYS165
		LYS149	2.25		
		ASN152	2.72		
		ASP147	2.79		
		ASP166	2.55		
		LYS149	2.93		
		ASP166	2.84		
	PLN	LYS51	2.61	3	CYS165, ILE28, TYR100, VAL36, ALA49, ILE28, VAL36, ALA49, LEU154, CYS165, VAL36
		GLY31	2.61		
		LYS51	2.48		
	BLP	SER32	2.72	6	ALA49, LEU154, VAL36, LYS51, LEU98, CYS165, TYR100, VAL36, ILE28, LEU154
		PHE33	2.15		
		ASP166	2.92		
		GLN151	2.60		
		ASP166	2.95		
		ASP99	3.52		
	STM	-	-	-	ALA49, ARG107, VAL76, LEU98, LEU154, CYS165, TYR106
	GNK	LYS51	2.81	3	VAL36, VAL36, ILE28, VAL36, ALA49, LEU154, CYS165
		VAL101	2.29		
		ASP99	2.61		
**1UDT**	VIA	TYR76	2.26	4	VAL246, MET280, PHE284, PHE284, PHE250, ALA231, VAL246, ALA247, ILE277, TYR76, TYR76, HIS77, LEU268, LEU229, VAL246
		GLN281	2.15		
		GLN281	1.83		
		TYR128	2.13		
	ASN	TYR76	1.90	6	PHE284
		GLN239	1.84		
		LEU229	2.99		
		GLN281	2.31		
		LEU229	3.68		
		MET280			
	ANN	TYR76	2.52	7	PHE284, PHE250, LEU229, VAL246
		ASP228	2.40		
		ASP228	2.55		
		GLU146	2.13		
		THR187	2.46		
		LEU229	1.97		
		TYR76	2.28		
	AXN	LEU189	2.23	3	PHE284, PHE284, HIS77, VAL246, VAL246
		LEU189	2.28		
		ASP188	2.52		
	STM	-	-	0	PHE284, LEU189, LEU189, MET280, LEU268, MET280
	STG	HIS121	1.59	2	LEU189, MET280, PHE284
		ASP188	2.21		
**2Y9Q**	ANP	LYS54	2.36	16	VAL39, ALA52, LEU156, ILE31, VAL39, ALA52, LEU156
		LYS151	1.94		
		ALA35	1.89		
		TYR36	2.42		
		TYR36	3.09		
		GLY37	2.04		
		LYS54	2.29		
		ARG67	2.58		
		ARG67	2.88		
		MET108	2.21		
		LYS151	2.95		
		GLN105	1.93		
		ASP106	2.20		
		SER153	1.85		
		GLY34	2.33		
		MET108	3.25		
	ASN	ALA35	1.84	10	-
		TYR36	2.50		
		LYS114	2.27		
		ASP106	2.88		
		GLY37	2.22		
		ASP167	2.22		
		GLY32	2.50		
		GLY37	2.64		
		LYS114	2.89		
		ASP167	3.15		
**2Z5X**	HRM	GLY67	2.59	2	TYR407, TYR407, TYR444, TYR407, MET445, TYR69 PHE352
		ASN181	3.71		
	7ML	ALA68	2.51	5	CYS406, MET445, ARG51, ALA448
		MET445	2.83		
		MET445	2.35		
		TYR69:O	2.64		
		TYR444	2.38		
	AGT	ALA68	2.50	3	CYS406, MET445, ARG51, ALA448
		MET445	2.83		
		TYR444	2.37		
	ASN	ALA111	2.72	12	HIS488
		ALA111	2.57		
		ALA111	2.76		
		PHE112	2.37		
		TYR121	2.23		
		ASP132	2.16		
		PHE112	2.98		
		GLU492	2.82		
		SER209	2.30		
		GLY110	2.77		
		ARG129	2.54		
		PRO113	3.30		
	GNK	ASN181	2.52	2	TYR407, TYR407, TYR407, ARG51
		TYR407	3.75		
	IFG	ALA68	2.23	5	CYS406, TYR407 TYR444, GLY66, GLY67
		TYR69	2.55		
		ASN181	2.12		
		ILE207	2.60		
		TYR444	3.27		
	IVA	GLY67	2.31	2	CYS406, GLY66, GLY67, ILE23, ARG51, ALA448
		TYR407	2.61		
	LRS	TYR69	2.98	3	MET44, TYR407, ARG51; THR52, ALA448, TYR407, TYR444, ILE23, ARG51, ALA448
		TYR407	2.98		
		GLY443	3.71		
	PLN	ALA68	2.17	6	TYR407, TYR407, TYR444, TYR407, MET445, ILE335 LEU337
		TYR444	2.68		
		MET445	2.65		
		TYR69:O	2.21		
		ILE180:O	2.47		
		ASN181	2.62		
**4AU8**	Z3R	ASP87	2.18	3	CYS84, VAL19, PHE81, VAL19, ALA32, VAL65, LEU134, ALA144, ALA32, LEU134
		ILE11	1.75		
		GLN86	2.59		
	AXN	ILE11	1.82	3	PHE81, ILE11, VAL19, ALA32, LEU134, ILE11, LEU134, VAL19, ALA32, LEU134, ALA144
		ILE11	2.43		
		LYS34	2.97		
		ASP85	3.06		
	AGR	CYS84	1.92	4	PHE81, PHE81, VAL19, ALA32, ALA144, ILE11, VAL19, LEU134
		ILE11	2.77		
		PHE83	2.62		
		CYS84	3.61		
	GNK	ASP85	2.83	3	VAL19, PHE81, ALA144, VAL65, LEU134, ALA32, LEU134, ILE11, VAL19, LEU134, ILE11
		ASP145	2.92		
	NKT	-	-	0	PHE81, PHE81, VAL19, ALA32, LEU134, ALA144, ALA144, VAL65
**4BDS**	THA	-	-	0	TRP79, TRP79, TRP79, TRP79, HIS435, ALA325, TRP79, TRP427
	AGT	TYR329	2.88	4	TRP228, TRP228, PHE326, PHE326, LEU283
		TRP427	2.75		
		HIS435	3.08		
		HIS435	2.62		
	ASN	GLY114	2.98	10	-
		GLY114	2.73		
		ALA196	3.06		
		SER284	2.31		
		SER195	2.83		
		GLY113	2.90		
		GLY114	2.55		
		LEU283	2.85		
		HIS435	3.28		
		TRP228	3.60		
	ANN	THR281	2.74	5	TRP79, TRP79, ALA325
		HIS435	2.65		
		TYR329	2.11		
		SER76	2.25		
		HIS435	2.51		
	AXN	ASP67	2.29	3	TRP79, TRP79, GLY112; GLY113
		SER195	3.04		
		GLU194	2.38		
	PLN	ASP67	2.38	3	ASP67, TRP79, TRP79, TRP79, TRP79, ALA325, MET434, TRP79, TRP427, TRP427, TYR437
		ASN65	2.66		
		GLY113	2.72		
	BLP	ASP67	3.07	3	TRP79, TRP79, HIS435, TRP79, TRP79
		GLY112	3.23		
		ASN80	3.41		
	DHK	-	-	0	TRP79, TRP79
	IFR	PRO282	2.08	1	TRP79, TRP79, TYR329
	IFG	GLY114	2.75	5	TRP79, TRP79, HIS435, TYR329
		TYR329	2.95		
		SER284	2.46		
		GLU194	2.54		
		GLY436	2.82		
	IVA	TRP79	2.31	4	TRP79, TRP79
		GLY75	2.58		
		ALA325	2.94		
		TRP79	2.63		
	LRS	TYR125	2.86	4	TRP79, TRP79, TYR329, LEU122, TRP79, TRP79, TYR329
		PRO282	2.49		
		GLU194	2.24		
		TRP79	3.63		
	STG	-	-	0	TRP79, ALA325, TRP79, TRP79, PHE326, TYR329, TYR329, HIS435
	STM	-		0	ALA325, TRP79, PHE326, TYR329, TYR329
**4KKE**	AMP	LYS49	3.04	11	VAL34, MET102, MET102, ALA47, VAL152, LEU162, ALA47, VAL152
		LYS49	4.72		
		ASN108	2.58		
		ASN108	2.06		
		ASN150	2.32		
		GLU103	2.57		
		MET102	2.88		
		SER149	2.32		
		GLY27	2.50		
		GLY29	2.61		
		LYS49	2.64		
		MET105	3.30		
	ASN	ASN108	2.13	7	-
		ASN108	2.31		
		GLN111	2.42		
		SER28	3.08		
		ASP106	2.75		
		GLY27	2.88		
		GLN111	2.90		
	ANN	ALA30	2.32	8	ALA30, VAL181, TYR179, LEU162, VAL34
		GLN31	2.22		
		ASP145	1.74		
		LEU162	2.14		
		ASP1632	2.93		
		GLY29	2.58		
		GLY29	2.70		
		GLN31	3.60		
	AXN	ALA30	2.28	8	VAL34, VAL34, LEU162, ILE26
		ASN108	2.02		
		ASN150	2.72		
		SER149	2.47		
		MET105	2.82		
		ASP106	2.93		
		GLY29	2.44		
		LYS49	2.58		
**4M0F**	1YK	TRP283	2.70	4	TRP283, TRP283, TRP283, TYR338, VAL291, TYR69, TYR121, TRP283, PHE294, PHE335, PHE335, TYR338
		HIS284	2.71		
		PHE335	3.46		
		ASP71	3.29		
	AGT1	HIS444	3.06	1	TRP283, TYR334, TYR338
	ASN	ASP71	2.64	10	-
		GLY123	2.81		
		TRP83	2.99		
		TYR121	2.59		
		TYR69	2.21		
		SER122	2.60		
		SER122	2.05		
		GLY123	2.83		
		TRP83	3.51		
		ASN84	3.02		
	ANN	SER290	2.74	6	TRP283, TYR338, TYR121, TYR334
		PHE292	2.99		
		PHE292	2.58		
		SER290	2.07		
		PHE292	3.00		
		TYR338	3.31		
	AXN	GLU199	2.29	3	TRP83, TRP283, TYR121, TYR121
		GLY118	2.45		
		TYR121	2.75		
	PLN	PHE292	1.92	4	TYR338, TRP83, TRP283, TRP283, TRP283, TRP283, TYR338, TYR338, TYR338, TRP83, TRP283, TYR334
		SER290	2.55		
		VAL291	2.36		
		PHE292	3.04		
	7ML	SER200	2.82	2	TRP283, TYR334, TYR338
		GLU199	2.85		
	BLP	SER200	2.30	5	TRP83, TYR338, TYR334, TYR338
		PHE292	2.43		
		ARG293	2.28		
		GLY123	3.08		
		GLY123	2.76		
	DGN	-	-	0	TYR334, TRP83, TRP83
	DHK	GLY118	3.04	3	TYR334, TYR338, PHE335, TYR338
		GLY119	2.72		
		SER200	2.53		
	GNK	HIS444	2.49	2	TRP83, TYR334, TYR334, TYR338, TRP83, TRP83, HIS444
	IFR	TYR121	2.69	4	TRP83, TYR334
		THR80	2.48		
		SER200	3.08		
		HIS444	2.39		
	IFG	TYR121	2.45	3	TRP83, TRP283, TRP283, TYR334, TYR338
		SER122	2.97		
		GLY117	2.09		
	IVA	THR80	2.72	5	TRP83, TRP83, PHE335, TYR334, TYR338, TRP83, TRP83, TYR334
		TRP83	2.58		
		GLY118	2.38		
		GLY118	2.74		
		SER122	3.43		
	LRS	GLU199	2.15	2	TRP83, TRP83, TYR338, VAL291, TRP83, TRP83, PHE335
		PHE335	3.31		
	STM	GLU199	2.32	3	TYR334, TRP83, TRP83, TRP283, TRP283, TYR338
		GLY445	2.70		
		GLY445	3.06		
**5BTR**	STL	ARG303	2.33	3	PHE271, VAL302, PRO69
		ASP149	2.27		
		LYS301	2.03		
	ASN	ARG303	2.24	4	-
		ASP149	2.17		
		THR66	2.89		
		GLY272	2.83		
	ANN	ASN12	2.82	8	HIS328, PRO327, HIS328 LEU63
		ASN12	2.10		
		ASN12	2.97		
		LYS90	2.80		
		ARG303	2.98		
		ILE84	1.97		
		GLU87	2.47		
		LEU85	2.66		
	STG	PRO304	2.62	1	PRO69, PHE271
	STM	-	--	0	PHE271, PHE271, PRO69, ILE80, ARG303, ARG303
**5IE1**	6BS	ASP34	2.11	4	TYR73, TYR73, TYR73, PHE110, VAL71
		ASP230	2.70		
		ASP230	2.18		
		GLY36	2.35		
	ASN1	TRP78	2.22	4	-
		THR331	2.52		
		ARG130	2.56		
		THR331	2.94		
**5LUA**	5KN -4.7	SER191	3.26	1	TYR203, CYS164, ALA193
	STM -6.5	-	-	0	TYR196, TYR196, TYR16, TYR16, TYR196
	7ML -6.4	SER191	2.28	2	TYR16, TYR203, ALA193
		TYR192	2.87		
	AGT -6.5	ALA193	2.16	4	TYR16, TYR203, TYR203, ALA193
		ALA193	2.55		
		CYS164	2.87		
		HIS123	3.08		
	ANN -6.7	TYR16	2.83	10	TYR16
		ASN17	2.62		
		ASN17	2.88		
		ASN17	2.71		
		ASN13	2.05		
		PRO132	2.06		
		PHE131	2.34		
		GLU134	2.96		
		ASP135	2.19		
		GLY14	2.82		
	ASN -6.4	ARG19	2.93	3	-
		TYR203	2.72		
	AXN -6.4	ARG19	2.41	4	TYR16, TYR16, TYR16
		ARG19	2.37		
		TYR196	2.25		
		ASN17	3.72		
	IVA -6.6	ALA193	2.57	3	TYR16, ALA193
		TYR192	2.66		
		TYR192	2.84		
	PLN	ALA193	2.07	3	HIS20, ALA193, CYS164, ALA193, ALA193
		TYR192	2.55		
		ASP122	2.98		
	IFR	ASP206	2.80	2	TYR203, ALA193, CYS164, ALA193
		ASN17	2.52		
	GNK	TYR196	2.57	3	TYR16, ALA193, ALA193
		SER201	2.28		
		SER191	3.68		
**6GZM**	LCI	ASP91	2.10	5	MET82, ILE15, ILE23, ALA36, LEU85, LEU135, ILE23, ILE148
		LEU85	1.92		
		LEU85	2.31		
		GLU83	3.28		
		SER17	3.52		
	ASN	LYS38	1.94	11	-
		LYS130	2.29		
		GLU52	2.45		
		ASP91	2.08		
		ASP132	2.80		
		SER88	1.93		
		LYS130	3.03		
		LEU85	3.48		
		GLY86	3.78		
		SER17	2.75		
	AXN	LEU85	2.30	3	ILE23, ALA36, LEU135, ILE148, ILE15, ALA36, LEU135, ILE23, ILE148
		GLY18	2.72		
		PRO87	2.59		
	GNK	ASN133	1.83	4	ILE23, ILE148, ILE23, ALA36, MET82, LEU135, ILE148
		GLY18	2.72		
		GLY18	2.41		
		LEU84	2.71		
	STM	-	-	0	ALA36, MET82, LEU85, LEU135, ILE148, ILE23, MET82, ILE148

ASN-Aquilarisin; AXN-Aquilarixanthone; ANN-Aquilarisinin; PLN-pilloin; AGT-agartetrol; STM-stigmasterol; STG- stigmastrol glycoside, GNK-genkwanin, IVA-isovannillic acid; 7ML-7-methyl luteolin; AGR-agarol, IFR-iriflophenone 2-O-rhamnoside, IFG-irflophenone 3-C-glucoside, LRS-laricirsenol, DHK-Dihydrokaranone, NKT-noorkatone, BLP-Balanophonin, DGN- delta-guiene, DK1-5,7-Dichloro-4-hydroxyquinoline-3-carboxylic acid; TMU- N-(4-methoxybenzyl)-N'-(5-nitro-1,3-thiazol-2-yl) urea; VIA- viagra; ANP- phosphoaminophosphonic acid-adenylate ester; HRM- Harmine; Z3R- 4-(1,3-benzothiazol-2-yl)thiophene-2-sulfonamide; THA-Tacrine; AMP-adenosine monophosphate; 1YK- territrem B; STL- resveratrol; 6BS- 3-[2-amino-6-(2-methylphenyl)quinolin-3-yl]-N-(3,3-dimethylbutyl)propenamide; 5KN-2,4-di (morpholin-4-yl)aniline; LCI- [4-[[4-[5-(cyclopropylmethyl)-1-methyl-pyrazol-4-yl]-5-fluoranyl-pyrimidin-2-yl]amino]cyclohexyl]azanium.

### Structural stability of the best docked complexes using MD

We selected 7 complexes of agarwood compounds 1Q5K-AXN (complex1), 2Z5X-PLN (complex2), 4BDS-ANN (Complex 3), 4M0F-AXN (Complex 4), 5IE1-ASN (Complex 5), 5LUA-AGT (Complex 6), and 1PBQ-ASN (Complex 7) having least binding energy towards selected AD targets. MD simulation quality check was performed over all the 7 trajectories by plotting temperature, pressure, and potential energy. During the 100ns simulation, the temperature and pressure were held constant at 300 K and 1 bar, respectively. We observed less fluctuations in the potential energy of the simulated systems, suggesting the well equilibration of all the complexes during simulation. In order to better understand the structural stability, the root mean square deviation (RMSD), root mean square fluctuation (RMSF), radius of gyration (Rg), and solvent accessible surface area (SASA) were also measured.

***Root mean square deviation (RMSD) analysis***

**Figure 4.**
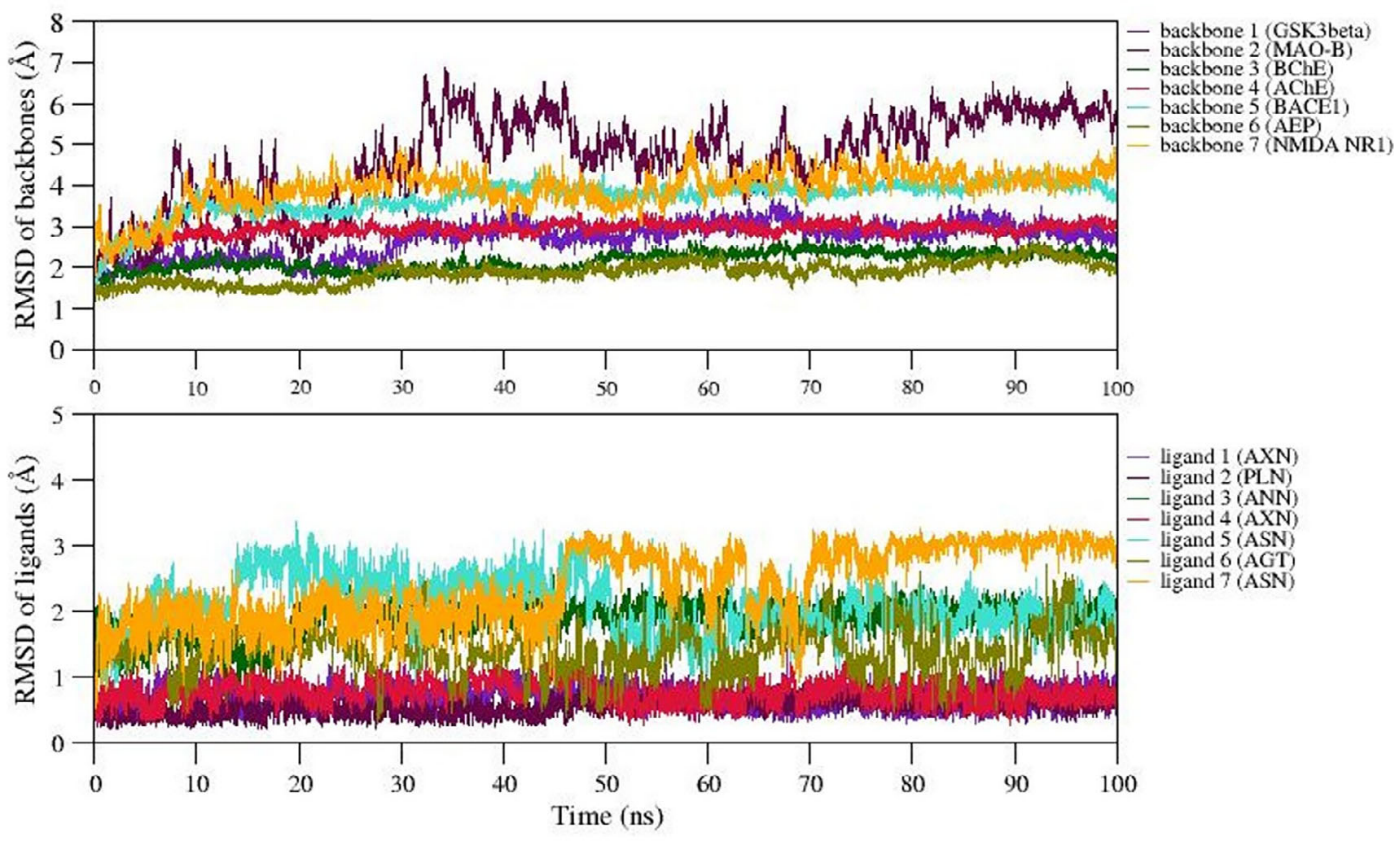
RMSD of A) backbone and B) ligands of selected complexes.

The backbone RMSD values were plotted over the trajectories revealing the stable dynamics expressed by all the simulated complexes during the 100ns. Figure 4 represents the backbone RMSD values of GSk3beta (indigo), BChE (Green), AChE (crimson), BACE1 (turquoise), and AEP (olive) showing moderate fluctuations up to 30ns (equilibration period), however, RMSD values are well stabilized after 50ns for these five proteins followed by equilibration period of 30ns. However, the RMSD of backbone atoms of NMDA (orange) and MAO-B (maroon) reached equilibrium after 60ns and 80ns during simulation. The average backbone RMSD values of GSk3beta, BChE, AChE, BACE1, AEP are observed as 2.5Å, 3.2Å, 2.1Å, 2.5Å, 2.4Å, etc. On the other hand, average RMSD values of NMDA, and MAO-B are found to be ~3.2Å and ~3.6Å, respectively. The representative MD simulation end structure extracted from the trajectory is compared with the initial starting MD simulation of the respective complexes. The analysis reveals the RMSD of structural superimposition of MD starting structure (0 ns) and end structure (100 ns) for studied 7 complexes has been found to be 1.339Å, 1.186Å, 1.122Å, 1.314Å 1.230Å, 1.161Å, 1.146Å. The ligand RMSD values also follows the similar trend (i.e., RMSD values <5Å) for all the ligands, however, the ligand7 (ASN) represents structural changes due to existence of torsions, which trigger the diverse ASN conformations during simulation. Thus, in general, all the 7 complexes express stable dynamics during 100ns MD simulation with backbone and ligand RMSD value of <5Å (refer Figure 4A and B).

***Root mean square fluctuation (RMSF) Analysis***

**Figure 5.**
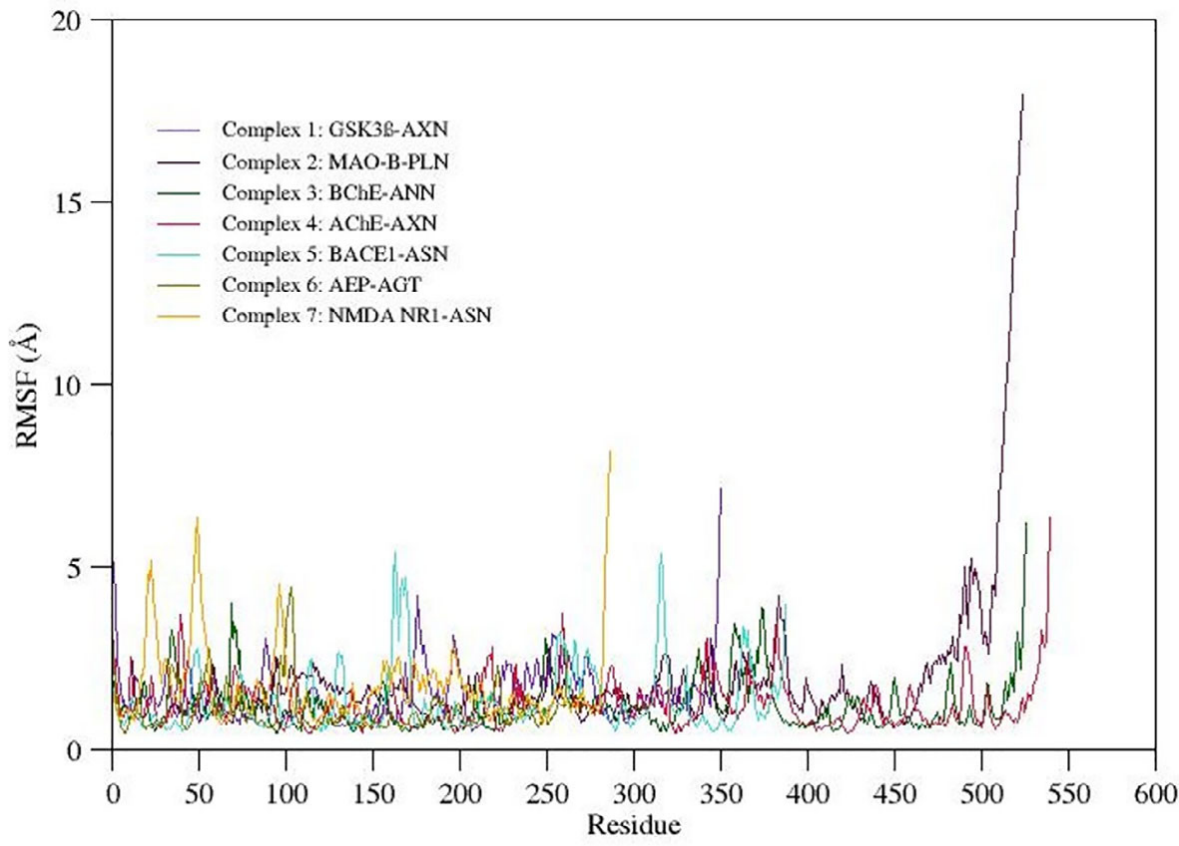
RMSF of selected complexes.

RMSF gives qualitative measure provides detailed insights to the conformational flexibility of protein structure. The RMSF value plotted for the C-alpha atoms of all the simulated systems is shown in Figure 5. The C-terminal region in all the complexes show highest fluctuations when compared to other regions in the protein structure. Careful observation of the three-dimensional structure reveals that increased RMS fluctuation values at C-terminal region are mainly due to the absence of native folded secondary structure. Overall binding pocket residues show the least RMS fluctuations (<3Å) as they actively participate in the stable non-bonded contacts. Also, other flexible loops and N-terminal regions express moderate to high fluctuations in the RMSF values. Due to a maximum residual fluctuation of up to 5, a greater peak between 175 and 190 amino acid residues was detected in complex 1. Only the C- and N-terminal portions of complex 2 exhibit persistent amino acid variation. Two higher peaks are observed in complex 3; one between 370 and 390 and one between 75 and 90 amino acid residues. Three higher peaks in complex 4 have been noticed at positions between 370 and 390 amino acid residues, between 250 and 275 amino acid residues, and between 40 and 60 amino acid residues (3.8Å, 3.9Å, and 3.8Å, respectively). Between 150 and 200 amino acid residues and 300 and 350 amino acid residues, complex 5 shows two higher peaks (each 5.5Å). A higher peak (4.5Å) has been seen in complex 6 between 90 and 110 amino acid residues. Three higher peaks (at 5Å, 6.5Å, and 4.5Å) have been discovered in complex 7, two of which were between 0 and 60 amino acid residues and one between 80 and 110.

***Analysis of Radius of Gyration (Rg) and solvent accessible surface area (SASA)***

**Figure 6.**
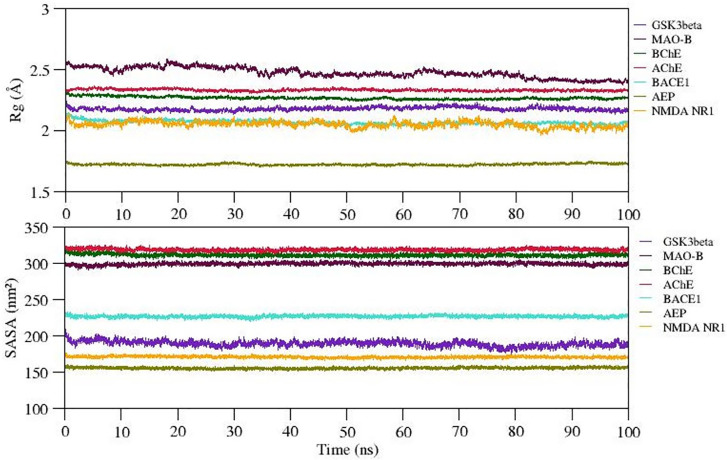
Compactness of docked complexes and exposure of hydrophobic pocket to the solvent estimated by Radius of Gyration (Rg) and solvent accessible surface area (SASA) respectively.

The radiation of gyration (Rg) explains the protein folding/compactness of the molecule, hence we analyzed the compactness of the protein-ligand complexes and exposure of hydrophobic core of the protein to the solvent upon ligand binding. The variation in Rg and SASA of selected complexes is given in Figure 6. The Rg value for complexes 1-7 (except complex 2) is well stabilized while complex 2 shows steady decrease in the Rg value during the MD simulation. Also, SASA values of all complexes show significant structural stability and represent the formation of compact globular shape during 100 ns simulation. The average Rg and SASA values of complexes 1 to 7 range between ~1.75 to 2.5Å and 155 to 320 (nm2). Thus, we observed stable complex formation in all the complexes (refer Figure 6).

***Intermolecular interactions observed in docked complexes***

**Figure 7.**
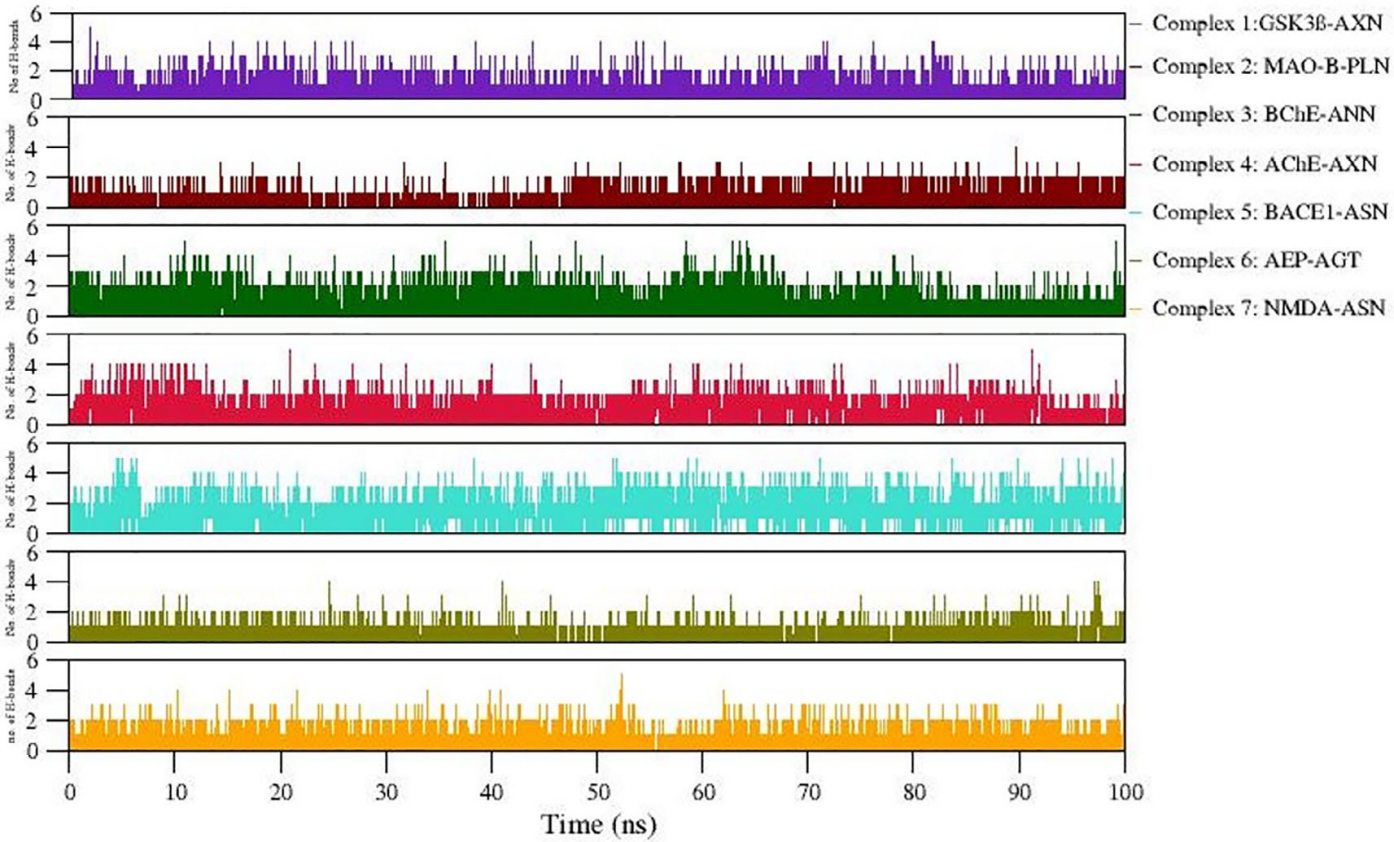
Number of H-bonds formed in 7 docked complexes during MD simulation.

**Figure 8.**
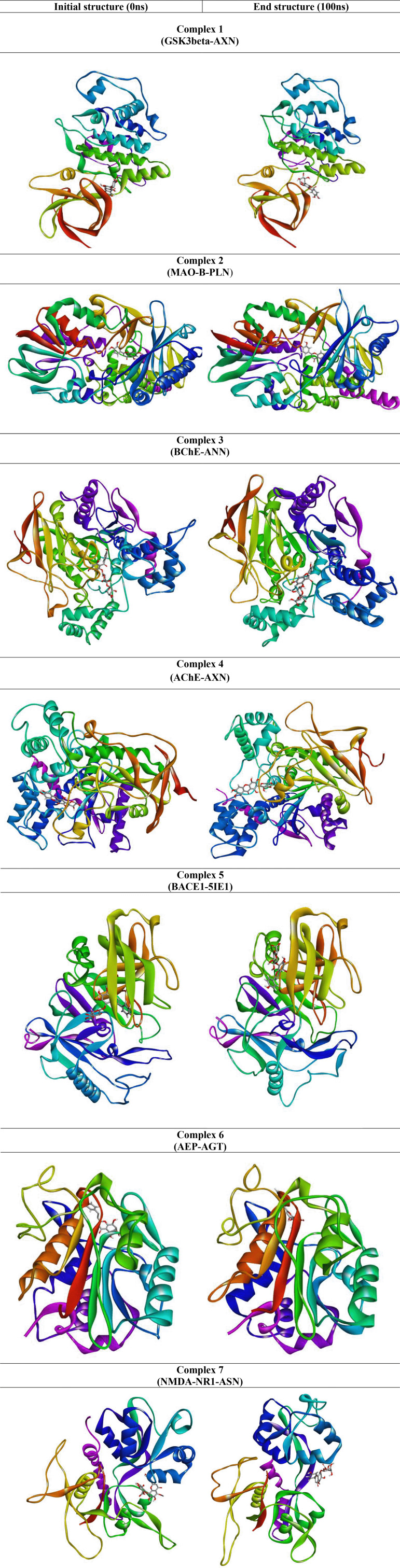
The representative snapshot of the initial and final structure of the MD simulation.

Formation of H-bonds between selected protein-ligand complexes has been monitored during 100 ns simulation. Figure 7 denotes the number of H-bonding interactions formed during simulation. Table 4 lists comparative analysis of non-bonded interactions observed in the initial starting structure (0ns) and MD simulation end structure (100ns). In general, the native non-bonded contacts were well conserved even after 100 ns MD simulation, revealing relatively more stable complex formation in all the simulated complexes. Hydrophobic interactions equally contribute in stabilizing these complexes. Moreover, the consistency of the observed H-bonds also supports the stable complex formation during simulation (Refer Figure 7). The snapshot of initial and final MD structure is shown in Figure 8, revealing the binding of ligands to the conserved binding site of their respective targets. Also, most of the complexes show compact folding during simulation forming much compact globular structure.

**Table 4.** Comparative analysis of nonbonded interactions of initial (0ns) and final (100ns) MD.

	Initial structure (0ns)		Final structure (100ns)	
	H bonds	Hydrophobic	H bonds	Hydrophobic
Complex 1 (1Q5K)	VAL101, ILE28, VAL101, ASP99, GLY29	LEU154, TYR100, VAL36, ILE28, ALA49	ARG107, PHE33, TYR100, VAL101, ASN30	VAL36, LEU154, ILE28
Complex 2 (2Z5X)	TYR69, ASN181	TYR407, TYR407, TYR407, GLY67, ALA68, MET445, ILE335, LEU337, MET350	ALA68, THR408, ASN181	PHE352, TYR407, TYR407, TYR444, TYR444, ILE23, MET445, LEU337, TYR69, PHE352
Complex 3 (4BDS)	HIS435, TYR329, SER284, SER284	ALA325	TYR329, HIS435, GLN116, GLY280	TRP79, TRP79, TRP79, PHE326, TYR329, VAL285, ALA325, VAL328
Complex 4 (4M0F)	TRP83	TRP283, TRP283, TRP283, TYR338, TYR121	PHE292, GLU289, GLY339, VAL291	TYR74, TYR74, TYR74, LEU286
Complex 5 (5IE1)	ARG130, TRP78, TYR200, SER37, ASN39	TYR73, TYR73, VAL71, TRP78, TRP78, VAL71	SER38, SER38, ASN39, ARG130, LIG388, LYS67, SER38, ASN39	PRO72, VAL71, ILE128
Complex 6 (5LUA)	ALA193 TYR192	ALA193	-	TYR203 TYR16
Complex 7 (1PBQ)	ARG127, GLN140, ARG183, ARG183, ARG243, SER176 ARG183	TYR180	ARG183, SER244, ARG243, ARG183, ARG183	TYR180

***Estimation of Binding Free Energy using MMPBSA***

The binding free energy for all the simulated complexes has been quantitatively measured by using MMPBSA methods over the well equilibrated trajectory observed between 0 to 100 ns. Table 5 represents the energy components including molecular mechanics, van der Waal (vdW) interactions, electrostatic, polar and nonpolar energies that significantly contribute to the binding free energy. The estimated binding free energy for complexes 1 to 7 is -81.018 ± 61.364, -159.438 ± 10.190, -227.959 ± 13.745, -152.764 ± 15.897, -149.090 ± 16.646, -79.236 ± 19.623, and -146.796 ± 12.694 kJ/mol, respectively. It has been observed that binding free energy of protein-ligand complexes is significantly influenced by both electrostatic and vdW interactions.

**Table 5.** Summary of binding energies of seven complexes after MD simulation.

	van der Waal energy (kJ/mol)	Electrostatic energy (kJ/mol)	Polar solvation energy (kJ/mol)	SASA energy (kJ/mol)	Binding energy (kJ/mol)
Complex 1	-141.612 ± 61.943	-13.655 ± 8.026	89.417 ± 26.108	-15.167 ± 6.895	-81.018 ± 61.364
Complex 2	-212.674 ± 7.642	-6.903 ± 3.923	78.114 ± 6.411	-17.976 ± 0.811	-159.438 ± 10.190
Complex 3	-291.492 ± 11.716	-5.934 ± 3.392	96.328 ± 13.723	-26.860 ± 0.845	-227.959 ± 13.745
Complex 4	-179.549 ± 16.339	-12.621 ± 4.941	56.778 ± 8.569	-17.372 ± 1.346	-152.764 ±15.897
Complex 5	-203.620 ± 14.591	-20.609 ± 10.678	95.024 ± 19.590	-19.886 ± 1.190	-149.090 ± 16.646
Complex 6	-117.953 ± 13.022	-3.252 ± 3.148	54.352 ± 18.85	-12.384 ± 1.035	-79.236 ±19.623
Complex 7	-182.514 ± 20.042	-18.647 ± 5.393	73.852 ± 17.891	-19.486 ± 1.803	-146.796 ± 12.694

***Conformational changes at secondary structural level***

**Figure 9.**
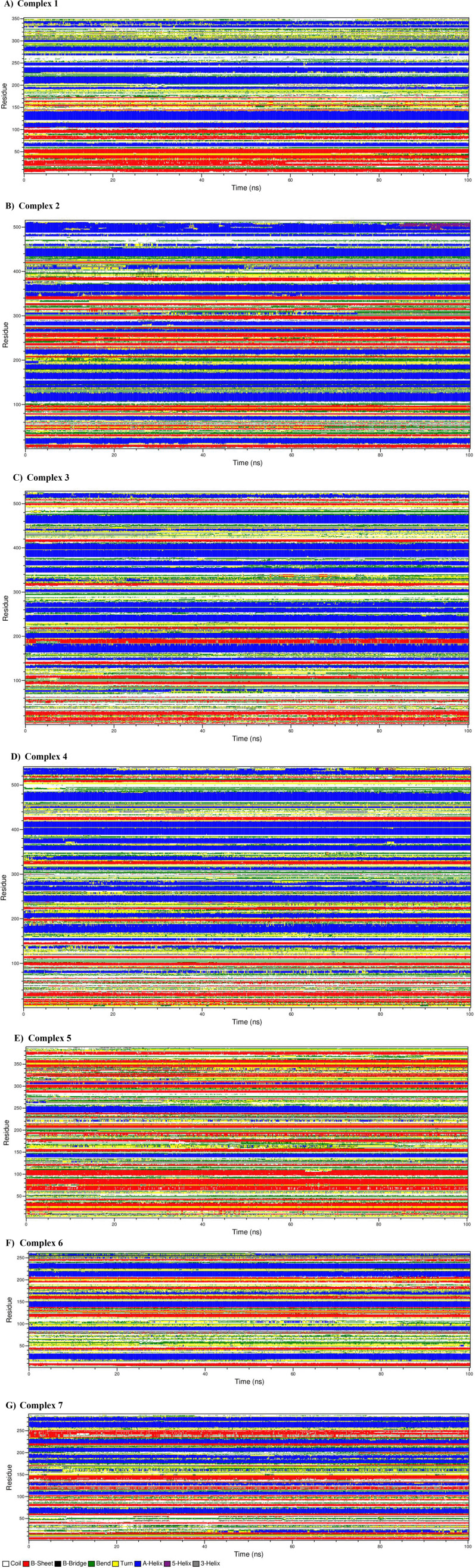
The dictionary of protein secondary structure elements. A) complex 1, b) complex 2, c) complex 3, d) complex 4, e) complex 5, f) complex 6, g) complex 7.

We further examined the secondary structural changes during simulation period using DSSP. The complexes 1-7 using DSSP plot have been shown as panel A-G in Figure 9. Various components of secondary structures are shown in specific colors as shown in figure legend. We noticed that major secondary structural components such as alpha helices and beta sheets are relatively much stable and express less variations in the secondary structure. Also, interestingly, the architecture of binding pocket is well maintained throughout the simulation due to rigidity provided by the alpha helices and beta sheets in all the simulated complexes. Thus, overall binding of ligand does not affect the secondary structure of protein. However, minor variations such as shortening, or extensions were noticed at flexible loop and linkers connecting the alpha helices and sheets. The H-bonds provide structural stability to the alpha helices and beta sheets in all the complexes.

### Enrichment analysis and Network construction

**Figure 10.**
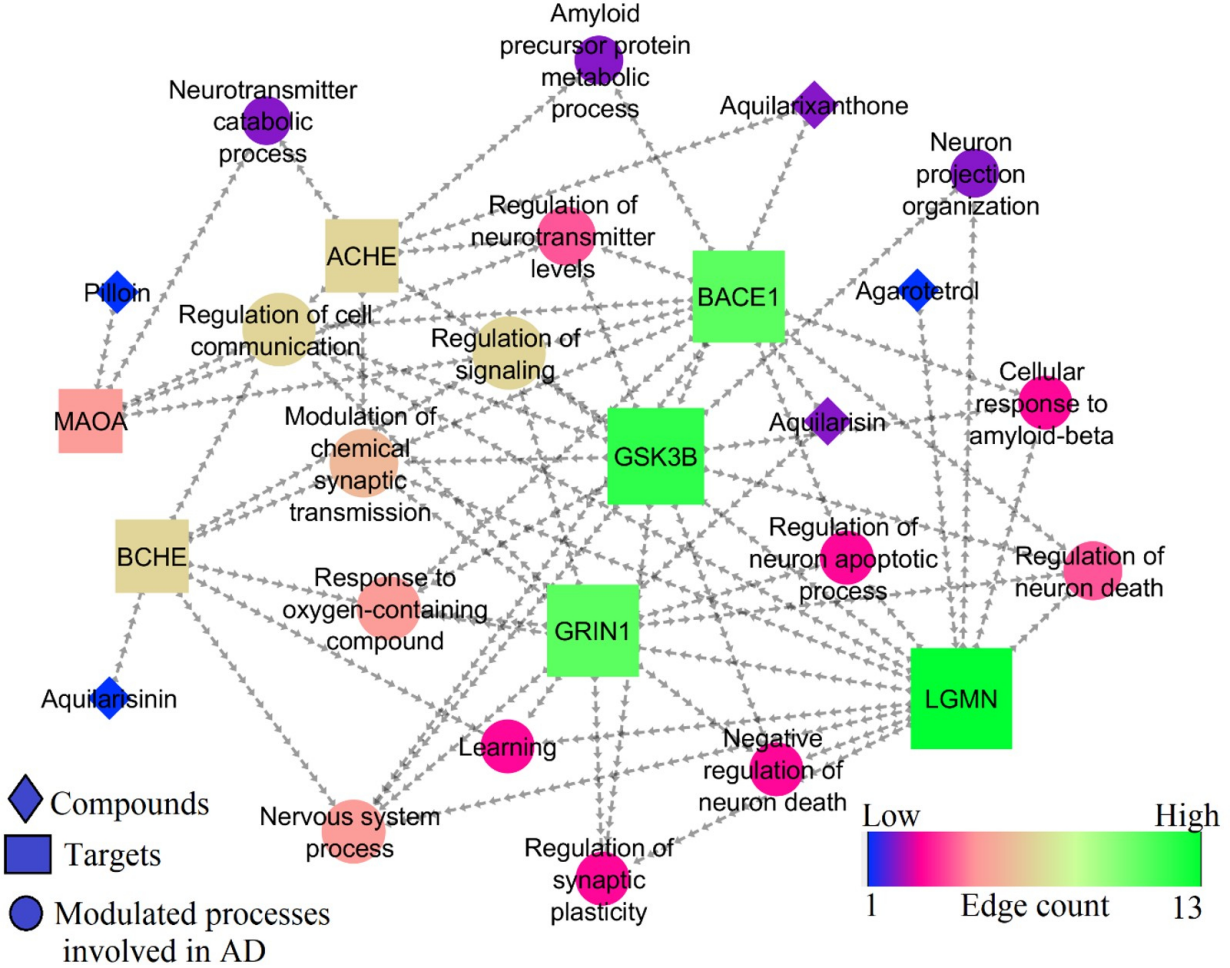
Network representation of compounds, targets, and modulated molecular processes involved in AD.

The GSK3B, MAOA, BCHE, ACHE, BACE1, LGMN, and GRIN1 all influenced 18 different biological processes. Among them, 15 were associated with AD. Among 7 targets, 6 targets *viz.*, BCHE, ACHE, BACE1, GSK3B, GRIN1, and LGMN were enriched for modulation of chemical synaptic transmission (GO:0050804) and 3 targets *viz.*, BACE1, GSK3B, LGMN were enriched for cellular response to amyloid-beta (GO:1904646). Following these, regulation of neurotransmitter levels, neuron death, cell communication, signaling, synaptic plasticity, neuron apoptotic process, learning, etc were also associated with SK3B, MAOA, BCHE, ACHE, BACE1, LGMN, and GRIN1 targets. Table 6 represents the biological processes modulated by best docked phytocompounds. Among the 7 targets, GRIN1, GSK3B, BACE1, and LGMN scored the highest edge count within the network of and were involved in multiple biological processes for the regulation of AD ( Figure 10).

**Table 6.** Gene Ontology process enrichment analysis describing the molecular processes involved in the AD.

GO ID	Description	Gene count	Background gene count	Strength	False discovery rate	Matching proteins within the network
GO:0050804	Modulation of chemical synaptic transmission	6	446	1.58	1.29E-05	BCHE, ACHE, BACE1, GSK3B, GRIN1, LGMN
GO:1904646	Cellular response to amyloid-beta	3	39	2.33	0.0014	BACE1, GSK3B, LGMN
GO:0001505	Regulation of neurotransmitter levels	4	231	1.68	0.002	ACHE, BACE1, GSK3B, MAOA
GO:1901214	Regulation of neuron death	4	317	1.55	0.0051	BACE1, GSK3B, GRIN1, LGMN
GO:0010646	Regulation of cell communication	7	3514	0.75	0.0111	BCHE, ACHE, BACE1, GSK3B, MAOA, GRIN1, LGMN
GO:0023051	Regulation of signaling	7	3553	0.74	0.0111	BCHE, ACHE, BACE1, GSK3B, MAOA, GRIN1, LGMN
GO:0042135	Neurotransmitter catabolic process	2	11	2.71	0.0111	ACHE, MAOA
GO:0007612	Learning	3	145	1.76	0.0155	BCHE, GRIN1, LGMN
GO:0042982	Amyloid precursor protein metabolic process	2	19	2.47	0.0211	ACHE, BACE1
GO:0050877	Nervous system process	5	1352	1.01	0.0254	BCHE, BACE1, GSK3B, GRIN1, LGMN
GO:0048167	Regulation of synaptic plasticity	3	191	1.64	0.0262	GSK3B, GRIN1, LGMN
GO:0043523	Regulation of neuron apoptotic process	3	212	1.6	0.033	BACE1, GRIN1, LGMN
GO:1901215	Negative regulation of neuron death	3	211	1.6	0.033	GSK3B, GRIN1, LGMN
GO:1901700	Response to oxygen-containing compound	5	1567	0.95	0.0372	BCHE, BACE1, GSK3B, GRIN1, LGMN
GO:0106027	Neuron projection organization	2	39	2.16	0.0478	GSK3B, LGMN

## Discussion

Aquilaria is an endangered agarwood-producing genus includes many plants species. Various parts of agarwood plants have been widely used as an important ingredient of traditional Ayurvedic, Chinese, Thai, Tibetan, and Eastern medicine. ^35^ Limited number of *in vitro* experiments have shown that the leaves, stem, and agarwood of Aquilaria plants, among other plant parts, exhibit neuroprotective properties. ^27^^,^^28^^,^^30^^,^^32^^,^^33^^,^^46^ Scientific investigation into the phytochemical components of agarwood is still quite limited because of the high industrial demand and loss of its natural resources. Therefore, it is crucial to carry out additional research investigations to develop premium goods and medications using agarwood's beneficial phytochemical components. In this regard, we carried out *in silico* studies to search for the agarwood hit molecules against the molecular targets of AD. The first step was to conduct docking studies to find the agarwood compounds with highest binding affinity against AD molecular targets. A total of five hit compounds (aquilarisin, aquilarisinin, aquilarixanthone, agarotetrol, and pillion) were identified from our docking results that demonstrated substantial binding affinity for several AD targets. Additionally, we chose these top seven docked complexes based on the binding energy values, the number and distance of hydrogen bonds, the number and distance of hydrophobic contacts, and conserved amino acid residues with native ligand interactions. We also chose two controls for comparative study. Total 7 systems were chosen and subjected for MD simulation study namely, complex 1: GSK3beta-AXN; complex 2: MAO-B-pilloin; complex 3: BChE-ANN; complex 4: AChE-AXN; complex 5: BACE1-ASN; complex 6: AEP-AGT, complex 7: NMDA-ASN).

A few of the theories proposed to explain the underlying molecular reasons of AD are the cholinergic theory, excitotoxicity, amyloid concept, and tau concept. According to the cholinergic theory, cognitive impairment in AD is caused by the loss of acetylcholine-synthesizing cholinergic neurons and consequent drop in ACh levels. ^1^ ACh is hydrolyzed to *acetyl coenzyme* A (acetyl CoA) and choline by two cholinesterases called acetylcholinesterase (AChE) and butyrylcholinesterase (BChE). In neuromuscular junctions and cholinergic synapses, ACh is mostly degraded by AChE rather than BChE under normal physiological circumstances. Cholinergic transmission can be stopped by AChE, a highly selective cholinesterase that can hydrolyze up to 25000 ACh molecules per second into acetate and choline. AD patients show up to a 67% decrease in the levels of AChE, while BChE levels rise to 120% of normal level. ^5^ This indicates that BChE could compensate for deficit in AChE by hydrolyzing ACh. The breakdown of acetylcholine at the synaptic cleft is prevented by cholinesterase inhibitors, which thereby improves cholinergic transmission. The current pharmacologically important cholinesterase inhibitors donepezil, galantamine, and rivastigmine could increase ACh levels in the brain and help in improving cognitive function. ^3^ However, Galantamine is the only naturally occurring inhibitor belonging to alkaloid class of phytochemicals and it can reversibly and competitively inhibit AChE. Therefore, it is very essential to identify potent cholinesterase inhibitors for the treatment of AD. Earlier, molecular docking studies showed that rutin (a flavone) showed improved AChE and BChE binding affinities compared to galantamine. ^47^ In this study we have shown that the best docked agarwood phytocompounds such as aquilarixanthone and aquilarisin express good binding affinity when compared to their known inhibitors of AChE and BChE, respectively. Most interestingly, we discovered aquilarixanthone had a higher binding affinity to AChE (-9.9 kcal/mol) than galantamine (-9.3 kcal/mol). Another factor contributing to neuronal death in AD is glutamate-induced excitotoxicity, which occurs when glutamate levels are too high and cause overstimulation of glutamate receptors such as the NMDA receptor. Memantine is the currently available NMDA receptor antagonist to normalize the glutamatergic system and ameliorate cognitive and memory deficits in AD. ^6^ The cleavage of amyloid beta (Aβ) from the amyloid precursor protein (APP) by the beta site amyloid precursor protein cleaving enzyme (BACE 1), also known as beta secretase, plays a role in the pathogenesis of AD. ^7^ As the inhibitors NMDA receptor and BACE1 are associated with reduction in glutamate and amyloid beta toxicity, respectively. Here we have observed stable interaction of aquilarisin with both NMDA receptor and BACE1. Asparagine endopeptidase (AEP), often referred to as human legumain, is known to have a role in the advancement of neurological illnesses such amyotrophic lateral sclerosis (ALS), ^48^ stroke, ^49^ and AD. It is also involved in a number of physiological functions, including immunological function. AEP is involved in the cleavage of amyloid precursor protein ^50^ and tau protein, ^51^ subsequently contributing to both amyloid and tauopathy in AD. This specifies the possible delta secretase activity of AEP. Previous studies have reported significantly high levels of AEP in the brains of AD patients as well as aged mice, suggesting the role of AEP in the onset and progression of AD. In view of this, targeting AEP may be useful for the amelioration of neurodegenerative disorders like AD. ^52^ Agarotetrol showed good binding affinity with the AEP in our study. Both monoamine oxidases A and B have been involved in the altered aminergic neurotransmitter levels seen in AD. ^53^ Activated MAO-A/B can destroy cholinergic neurons, induce amyloid β peptide production and accumulation, formation of neurofibrillary tangles and subsequent cognitive dysfunction. ^54^ Selegiline, an MAO inhibitor used to treat Parkinson's disease, has been tested for the treatment of memory impairment in AD. By preventing reactive astrocytes from producing gamma-aminobutyric acid (GABA), selegiline has been shown to enhance synaptic plasticity, learning, and memory in AD mice. ^55^ It has also been suggested that MAO-A inhibitor also offers neuroprotection. ^9^ Our docking studies also showed that pillion (-9.9 kcal/mol) has more affinity towards MAO-A compared to the native ligand as well as the control drug selegiline (-7.4 kcal/mol). The best docked agarwood compounds, such as agarotetrol, aquilarisin, and pillion are chromones, aquilarisinin is a chalcone, and aquilarixanthone is a xanthone, are complexed with the best docked complexes that we have chosen for MD simulation. Oxygen-containing heterocyclic compounds like chromones and xanthones are known for their antioxidant capabilities. Our *in silico* study suggests that compounds like agarotetrol, aquilarisin, aquilrixanthone, aquilarisinin, and pillion may be good lead candidates; however, further experiments studies would be required given that hydroxylated chromones and xanthones demonstrated reactive oxygen species (ROS) and reactive nitrogen species (RNS) scavenger effects. ^56^

In AD, twisting and tangles occur in the tau protein. As the tangles clump together, some nerve cells perish. This makes cell communication much more difficult. As connections between neural networks weaken, brain regions start to shrink. ^57^ Also, AD has a key pathogenic hallmark known as brain atrophy brought on by neuronal loss. Amyloid beta, which makes up the majority of senile plaques, is assumed to play a key role in the death of neurons and may play a role in synapse and neural network dysfunction as well as cognitive impairment in AD. ^58^^,^^59^ In the present study, phytocompounds were predicted to target GSK3B, MAOA, BCHE, ACHE, BACE1, LGMN, and GRIN1 and found to regulate neurotransmitter levels, cell communication, signalling, cellular response to amyloid-beta, learning, amyloid precursor protein metabolic process, nervous system process, regulation of synaptic plasticity and neuron apoptotic process. Also, these compounds modulate negative regulation of neuron death, response to oxygen-containing compounds, and neuron projection organization processes.

Agarwood plants are traditional medicinal plants which have been recently categorized as endangered and threatened plants. Considering the significant potential of agarwood in various health promoting effects and limited knowledge highlighting neuroprotective properties, we aimed to find best possible lead molecules for AD. We extensively used molecular modelling approach to screen the library of selected agarwood phytocompounds against key AD targets. The phytocompounds aquilarisin, aquilarisinin, and aquilarixanthone have great potential to inhibit multiple AD targets with the highest binding affinity. It is interesting to note that, these compounds express stable binding and conserved active site interactions when compared to their respective known inhibitors. Furthermore, a 100ns all-atom MD simulation in an explicit solvent was used to look at the structural stability and intermolecular interactions for some of the top found hits. During MD simulation, all the complexes reached equilibrium earlier than 30 ns and thereafter expressed stable dynamics throughout the simulation. The estimated binding free energy using MMPBSA approach for all the complexes shows that these phytocompounds show much better efficacy in successful inhibition of AD targets by fitting well into the cavity. They also express least fluctuations and form compact globular shape due to increased intermolecular non-bonded interactions during MD simulation. Notably, aquilarisin, aquilarisinin, and aquilarixanthone fail to pass the Lipinski rule, but still their bioactivity observed in our computational study is remarkable. However, further experimental studies (either in cell line or animal models) are essential to validate neuroprotective potential of these agarwood phytocompounds.

## Ethical Considerations

This study does not require any ethical approval as it does not involve any endangered species (plant or animal).

## Data Availability

Zenodo: In silico molecular docking and molecular dynamic simulation of agarwood compounds with molecular targets of Alzheimer's disease [Data set]. Zenodo. https://doi.org/10.5281/zenodo.7567232. ^60^ The Underlying Data for this Project are as Follows:
• Agarwood Compounds_PDB formats.zip • Graphical Abstract.jpg •
Proteins_structure_3D.zip Zenodo: In silico molecular docking and molecular dynamic simulation of agarwood compounds with molecular targets of Alzheimer's disease [Data set]. Zenodo. https://doi.org/10.5281/zenodo.7567232. ^60^ The extended data for this Project are as Follows:
• Supplementary Material ADMET properties of agarwood compounds.xlsx • Tables.docx Data are accessible in accordance with the provisions of the Creative Commons Attribution 4.0 International license (CCBY 4.0).
