# Supplementary material for: In silico molecular docking and molecular dynamic simulation of agarwood compounds with molecular targets of Alzheimer’s disease 
Source: F1000Res. 2024 Dec 16;12:230. Originally published 2023 Mar 1. [Version 2] doi: 10.12688/f1000research.130618.2 (PMC11809694; doi:10.12688/f1000research.130618.2)
Supplement: Peer review history

# Reviewer report: Reviewer response for version 2

**DOI:** 10.5256/f1000research.175226.r352655

**Published:** 2025-01-08

**Authors:** James Adams

**Affiliations:** 1 University of Southern California, Los Angeles, CA, USA

In silico molecular docking and molecular dynamic simulation of agarwood compounds with molecular targets of Alzheimer’s disease

 Alugoju P, Vishnu Bhandare V, S. Patil V, V. K. D KS, Borugadda PK and Tencomnao T

 Alugoju and coauthors have conducted a useful search for new compounds to treat AD. The methods are well described and reproducible including computer modeling of potential drug candidates. The compounds are found in endangered species that should not be harvested in large amounts for investigation or drug isolation. The interesting compounds found in this analysis can be synthesized for further examination. This study is a good example of how to investigate chemical compounds found in endangered species for use as drugs.

Is the work clearly and accurately presented and does it cite the current literature?

Yes

If applicable, is the statistical analysis and its interpretation appropriate?

Yes

Are all the source data underlying the results available to ensure full reproducibility?

Yes

Is the study design appropriate and is the work technically sound?

Yes

Are the conclusions drawn adequately supported by the results?

Yes

Are sufficient details of methods and analysis provided to allow replication by others?

Yes

Reviewer Expertise:

traditional medicine

I confirm that I have read this submission and believe that I have an appropriate level of expertise to confirm that it is of an acceptable scientific standard.

# Reviewer report: Reviewer response for version 2

**DOI:** 10.5256/f1000research.175226.r350343

**Published:** 2025-01-03

**Authors:** Abdul Rajjak Shaikh

**Affiliations:** 1 University Parthenope of Naples,, Naples, Italy

The manuscript titled “In silico molecular docking and molecular dynamic simulation of agarwood compounds with molecular targets of Alzheimer’s disease” presents virtual screening, docking, and molecular dynamics studies aimed at identifying lead compounds for Alzheimer's disease derived from natural products of Agarwood. Given the potential neuroprotective properties of endangered medicinal plants like Aquilaria, this research is significant. The manuscript can be accepted following the incorporation of the modifications outlined below:

 1. Please include relevant references to support the statement, “Phytocompounds from the Aquilaria plant species were selected based on previous literature.”

 2. Verify the correct abbreviation for lipophilicity and solubility, as both are currently referred to as (Mol LogP).

 3. Clarify whether blind docking was performed or if an active site was defined. Additionally, please provide the grid center values used for docking these complexes.

 4. Ensure that abbreviations are used consistently throughout the manuscript (e.g., Caco-2 vs. Caco2).

 5. Clarify what is meant by H-bond length (Å). Is it between H-A? Additionally, Table 3 appears unnecessarily expanded; please make it more compact.

 6. Carefully check sentences for grammatical errors, such as “Aquilarisin showied good binding affinity.”

 7. In the discussion regarding structural stability, you mention selecting seven compounds with the least binding energy towards selected AD targets. Please clarify what is meant by "least binding energy" and consider rephrasing for clarity.

 8. Define what is meant by complex 1 and complex 2 etc. earlier in the manuscript, as they are only defined later in Table 4. Consistency in naming complexes should be maintained throughout.

 9. Please calculate interaction energy to analyze contributions from Coulombic interactions and Lennard-Jones forces.

Is the work clearly and accurately presented and does it cite the current literature?

Yes

If applicable, is the statistical analysis and its interpretation appropriate?

Partly

Are all the source data underlying the results available to ensure full reproducibility?

Partly

Is the study design appropriate and is the work technically sound?

Yes

Are the conclusions drawn adequately supported by the results?

Partly

Are sufficient details of methods and analysis provided to allow replication by others?

Partly

Reviewer Expertise:

Molecular Modelling, Drug Design, Molecular Dynamics, Molecular Docking, Virtual screening

I confirm that I have read this submission and believe that I have an appropriate level of expertise to confirm that it is of an acceptable scientific standard.

# Reviewer report: Reviewer response for version 1

**DOI:** 10.5256/f1000research.143388.r229488

**Published:** 2024-01-23

**Authors:** Yassir Boulaamane, Iman Touati

**Affiliations:** 1 Abdelmalek Essaadi University, Tetouan, Morocco

- Methods section in abstract should be more detailed.
- Introduction lacks experimental data (IC _50_, *Ki*…) on the described activities of Agarwood compounds.
- What is the rationale behind using the 13 selected protein targets?
- Recommended range for each chemical descriptor needs to be added in table 2.
- Data describing ADMET of tested ligands should be added in the manuscript instead of supplementary material.
- BBB permeability is a crucial parameter for Alzheimer’s drugs; however, this property was not taken in consideration.
- Abbreviation of ligands should be used in the beginning, so the reader doesn’t get confused halfway through.
- It’s not clear why complexes are exhibiting out of bound fluctuations outside the C- and N-terminal region in figure 5. RMSF section should be re-analysed taking into consideration the dynamic behavior of Apo forms of each protein.
- Authors failed to highlight the key interactions for each protein and how they are at the origin of selectivity for the native ligands and if they are implicated in the binding of Agarwood compounds.
- No experimental studies in the literature were linked to back up the final conclusive remarks.

Is the work clearly and accurately presented and does it cite the current literature?

Yes

If applicable, is the statistical analysis and its interpretation appropriate?

Not applicable

Are all the source data underlying the results available to ensure full reproducibility?

Partly

Is the study design appropriate and is the work technically sound?

Yes

Are the conclusions drawn adequately supported by the results?

Partly

Are sufficient details of methods and analysis provided to allow replication by others?

Partly

Reviewer Expertise:

Computer-aided drug discovery, natural products, QSAR modelling, neurodegenerative diseases, molecular dynamics simulations

We confirm that we have read this submission and believe that we have an appropriate level of expertise to state that we do not consider it to be of an acceptable scientific standard, for reasons outlined above.

# Response

**Published:** 2024-11-23

**Authors:** Tewin Tencomnao

**Affiliations:** Faculty of Allied Health Sciences, Chulalongkorn University, Thailand

Responses to Comments

- Methods section in abstract should be more detailed.

***Response:*** Thank you for highlighting the importance of including a more detailed methodology in the abstract. As suggested, we have incorporated a more detailed methodology in the revised version of the manuscript.

- Introduction lacks experimental data (IC _50_, *Ki*…) on the described activities of Agarwood compounds.

***Response:*** Thank you for insightful comment regarding the activities of agarwood compounds. While there is growing research on the therapeutic potential of Agarwood extracts, specific IC50 and Ki values for activity of agarwood compounds against Alzheimer's disease have not yet been reported in the literature.

- What is the rationale behind using the 13 selected protein targets?

***Response:*** ****Alzheimer's disease (AD) involves multiple pathways including amyloid-beta aggregation, tau phosphorylation, oxidative stress, inflammation, and neuronal cell death. Experimental evidence and animal studies revealed significant involvement of the 13 chosen target proteins. This information has been included in the introduction section of revised manuscript.

- Recommended range for each chemical descriptor needs to be added in table 2.

***Response*** *:* As per the reviewer’s suggestion, we have now included the recommended range for each chemical descriptor in Table 2 of the revised manuscript.

- Data describing ADMET of tested ligands should be added in the manuscript instead of supplementary material.

***Response:*** As suggested by the reviewer, we have included a detailed description of the ADMET properties of agarwood compounds in the revised manuscript. Please refer to “Prediction of ADMET analysis” section in the Results.

- BBB permeability is a crucial parameter for Alzheimer’s drugs; however, this property was not taken in consideration.

***Response: *** We appreciate the reviewer’s insightful comment on the importance of BBB permeability in Alzheimer's drugs. This study has indeed considered the necessity for agarwood compounds to cross the BBB to exhibit anti-Alzheimer's effects. Molecules with logBB > -1 were classified as BBB+, while molecules with logBB ≤ -1 were classified as BBB-. All compounds (agarotetrol, AGT, aquilarisin, ASN, aquilarisinin (ANN), aquilarixanthone, AXN, and pillion, PLN) used for MD simulations have logBB values greater than -1, so they are classified as BBB+. This information has been included in the “Molecular docking analysis” section of revised manuscript.

- Abbreviation of ligands should be used in the beginning, so the reader doesn’t get confused halfway through.

***Response: *** Thank you for your suggestion. We have included the abbreviations of ligands when they are first used in the beginning of the document.

- It’s not clear why complexes are exhibiting out of bound fluctuations outside the C- and N-terminal region in figure 5. RMSF section should be re-analysed taking into consideration the dynamic behavior of Apo forms of each protein.

***Response: *** Dear reviewer, we have revised the RMSF section and please refer to the revised Figure 5. We have explained the RMSF of each complex with respect to functional and non-functional domains. For example, in complex 2: the MAO-A protein structure C-terminal region (Ile471 to Leu524) showed increased RMSF to 20Å as this region appears to be an extended, peripheral loop, located away from the active site. This area showed higher RMSF due to its position on the protein’s surface, where it is more solvent-exposed and less constrained by interactions with other residues, making it inherently more flexible. Additionally, this region does not contribute directly to the catalytic function, so structural rigidity is less, unlike the core active site.

- Authors failed to highlight the key interactions for each protein and how they are at the origin of selectivity for the native ligands and if they are implicated in the binding of Agarwood compounds.

***Response*** ***: *** Dear reviewer, the key interactions for each protein at initial (0ns) and final (100ns) conformations were compared to identify the behavior and site-specific interaction of ligand throughout 100 ns simulation. This information is included in the “Intermolecular interactions observed in docked complexes” section of the revised manuscript. The 2D interaction figures are provided as Supplementary Figure 8a.

- No experimental studies in the literature were linked to back up the final conclusive remarks.

***Response:*** To date, there have not been experimental studies demonstrating the neuroprotective efficacy of the agarwood compounds (aquilarisin, aquilarisinin, aquilariaxanthone, pilloin, or agarotetrol) reported in the present study. The absence of experimental validation limits the ability to conclusively assert the therapeutic potential of these compounds in treating Alzheimer’s disease.

# Reviewer report: Reviewer response for version 1

**DOI:** 10.5256/f1000research.143388.r221667

**Published:** 2023-12-18

**Authors:** Sailu Sarvagalla

**Affiliations:** 1 Department of Cellular and Molecular Medicine, College of Medicine, University of Arizona, Tucson, Arizona, USA

Summary

 The main aim of this paper is to identify natural compounds as hits for AD disease. In this regard, authors explored the utility of Aquilaria plant spp. derived natural compounds as a potential hit for AD related drug targets. For this purpose, authors used routine computer aided drug design techniques to evaluate agarwood compound’s binding mode and interaction strength with different AD targets. The data depicted in this paper is valuable, and clearly demonstrated some of hits have comparable binding strength and interaction as of native ligands of AD targets. In my opinion, rationalization is good, but needs some sort of in vitro validation for their potential application as a hit to lead development.

Is the work clearly and accurately presented and does it cite the current literature?

Yes

If applicable, is the statistical analysis and its interpretation appropriate?

Yes

Are all the source data underlying the results available to ensure full reproducibility?

Yes

Is the study design appropriate and is the work technically sound?

Yes

Are the conclusions drawn adequately supported by the results?

Yes

Are sufficient details of methods and analysis provided to allow replication by others?

Yes

Reviewer Expertise:

Structural bioinformatics, drug design, proteomics and genomics

I confirm that I have read this submission and believe that I have an appropriate level of expertise to confirm that it is of an acceptable scientific standard.

# Response

**Published:** 2024-11-23

**Authors:** Tewin Tencomnao

**Affiliations:** Faculty of Allied Health Sciences, Chulalongkorn University, Thailand

Thank you for reviewing our manuscript. We genuinely appreciate your positive feedback and are delighted that you believe our work will resonate with scientists in the field. Experimental validation for these preliminary and interesting results, based on in silico studies, has already been planned and will soon be communicated as a separate manuscript. We acknowledge the need for further validation, and we plan to conduct in vitro studies to confirm the potential of these hits for lead development. Future work will focus on refining these candidates and exploring their biological activity to enhance our findings and support their applicability in drug discovery.
